# A Systematic Review of Immunological Studies of Erythema Nodosum Leprosum

**DOI:** 10.3389/fimmu.2017.00233

**Published:** 2017-03-13

**Authors:** Anastasia Polycarpou, Stephen L. Walker, Diana N. J. Lockwood

**Affiliations:** ^1^Faculty of Infectious and Tropical Diseases, Clinical Research Department, London School of Hygiene and Tropical Medicine, London, UK

**Keywords:** erythema nodosum leprosum, leprosy, type 2 reaction, immunology, systematic review, TNF-α, neutrophils, immune complexes

## Abstract

Erythema nodosum leprosum (ENL) is a painful inflammatory complication of leprosy occurring in 50% of lepromatous leprosy patients and 5–10% of borderline lepromatous patients. It is a significant cause of economic hardship, morbidity and mortality in leprosy patients. Our understanding of the causes of ENL is limited. We performed a systematic review of the published literature and critically evaluated the evidence for the role of neutrophils, immune complexes (ICs), T-cells, cytokines, and other immunological factors that could contribute to the development of ENL. Searches of the literature were performed in PubMed. Studies, independent of published date, using samples from patients with ENL were included. The search revealed more than 20,000 articles of which 146 eligible studies were included in this systematic review. The studies demonstrate that ENL may be associated with a neutrophilic infiltrate, but it is not clear whether it is an IC-mediated process or that the presence of ICs is an epiphenomenon. Increased levels of tumor necrosis factor-α and other pro-inflammatory cytokines support the role of this cytokine in the inflammatory phase of ENL but not necessarily the initiation. T-cell subsets appear to be important in ENL since multiple studies report an increased CD4^+^/CD8^+^ ratio in both skin and peripheral blood of patients with ENL. Microarray data have identified new molecules and whole pathophysiological pathways associated with ENL and provides new insights into the pathogenesis of ENL. Studies of ENL are often difficult to compare due to a lack of case definitions, treatment status, and timing of sampling as well as the use of different laboratory techniques. A standardized approach to some of these issues would be useful. ENL appears to be a complex interaction of various aspects of the immune system. Rigorous clinical descriptions of well-defined cohorts of patients and a systems biology approach using available technologies such as genomics, epigenomics, transcriptomics, and proteomics could yield greater understanding of the condition.

## Introduction

Leprosy is an infectious disease predominantly of skin and peripheral nerves, caused by the obligate, intracellular, acid-fast bacillus *Mycobacterium leprae*. The organism shows tropism for macrophages and Schwann cells (1). The pathology and clinical phenotype of leprosy is determined by the host immune response to *M. leprae* (2). Patients develop leprosy on a clinical spectrum ranging from tuberculoid leprosy through borderline forms to lepromatous leprosy (LL) of the Ridley–Jopling classification (2). Patients with tuberculoid leprosy have a strong cell-mediated immune response to *M. leprae* limiting the disease to a few well-defined skin lesions and/or peripheral nerves (3). Patients with LL have absent cellular immunity and high titers of antibodies against *M. leprae*, which are not effective in controlling the bacilli (4).

Multi-drug therapy (MDT) is highly effective for treating the infection (1). However, despite this, 30–40% of patients with leprosy undergo immune-mediated inflammatory episodes such as Type 1 reactions (T1R) and erythema nodosum leprosum (ENL or Type 2 reactions) (5).

ENL is a painful inflammatory complication occurring in 50% of LL patients and 5–10% of borderline lepromatous leprosy (BL) patients particularly those with a bacterial index above 4 (6), whereas T1R predominantly affect those with borderline tuberculoid leprosy (BT), mid-borderline, and BL leprosy. Individuals with ENL present crops of painful, erythematous skin nodules with systemic symptoms of fever and malaise (6). ENL is a multisystem disorder and other organ involvement includes iritis, arthritis, lymphadenitis, orchitis, and neuritis (6). The histology of ENL skin lesions often shows an intense perivascular infiltrate of neutrophils throughout the dermis and subcutis (7) and vasculitis with edema of the endothelium together with granulocyte infiltration of vessels walls (8–10). However, not all ENL skin biopsies show evidence of vasculitis (10–13).

ENL is usually treated with high-dose oral corticosteroids or thalidomide if it is available and affordable. High doses of clofazimine are also commonly used (6). Treatment often lasts for many months or years. Few patients experience a single episode of acute ENL with the majority experiencing recurrent or chronic disease (6, 14). Prolonged use of oral corticosteroids is associated with multiple adverse effects (6). Our group has demonstrated that ENL results in significant economic hardship, morbidity, and mortality in patients (15, 16).

ENL is often described as a neutrophilic immune-complex-mediated condition, while there is evidence that T-cells further complicate the immunopathology. Elevated levels of certain cytokines such as tumor necrosis factor (TNF)-α and other immunological factors have been associated with episodes of ENL.

**Figure 1:**
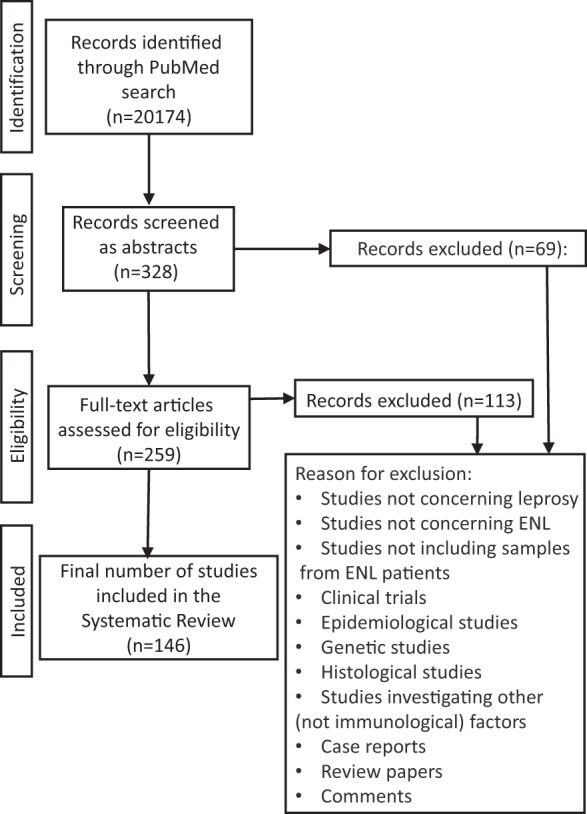
**Flowchart**. Flowchart of included studies.

We performed a systematic review of the published literature and critically evaluated the current evidence for the role of immunological factors that have been associated with the ENL. We created a flowchart showing our search strategy by identifying the studies to be included in this systematic review (Figure 1). We divided the systematic review into sections according to the immune parameter under investigation including neutrophils, immune complexes/complement, T-cells, and cytokines. Furthermore, we sought to identify possible methodological issues that might account for discrepancies between studies and to make recommendations for future immunological studies of ENL. The studies that we considered to have the most important findings are discussed in detail, while all the studies included in the review are summarized in the comprehensive tables.

## Methodology

The Preferred Reporting Items for Systematic review and Meta-Analysis Protocols (PRISMA-P) 2015 guideline was used to prepare this systematic review (17).

### Searching

Searches of the literature were performed up to 31st October 2016 in PubMed by the first author. Keywords used were: Hansen* OR Type 2 OR Type II OR leprosy OR lepra*, AND reaction OR erythema nodosum leprosum OR ENL. The references included in each study were also checked for potentially relevant publications.

### Inclusion Criteria

Immunological studies in PubMed, independent of published date, using samples from patients with ENL were included. Human samples including sera, peripheral blood mononuclear cells (PBMC), skin biopsies, or any other tissue were eligible for inclusion. Publications in languages other than English were translated.

An immunological study was defined as any study of the molecular and cellular components that comprise the immune system, including their function and interaction.

### Results of Search

The search in PubMed revealed 95,771 records, which were narrowed down by using restrictions, species (humans), and search fields (title/abstracts), leading to 20,174 records (Figure 1). A total of 19,846 studies were excluded by title because they did not address leprosy or ENL. Others were excluded because they did not include samples from ENL patients or they were clinical trials, epidemiological studies, case reports, review papers, commentaries, histological studies, genetic studies, and investigations of non-immunological factors. The abstracts of the remaining 328 titles were reviewed and a further 69 studies were excluded due to the same considerations.

The 259 papers were obtained full text of which 113 were excluded for the reasons described above. When there was doubt about studies, the first and second author agreed on whether they should be included in the systematic review. Data were extracted from the 146 eligible studies. Of these 146 eligible studies, 5 studies investigated the role of neutrophils in ENL, 28 studies investigated the role of immune complexes and complement in ENL, 44 studies investigated the role of T-cells in ENL, and 49 studies investigated the role of cytokines in ENL, of which 30 investigated the role of TNF-α in ENL. Sixty-four studies investigated the role of other immunological factors in ENL.

### Data Synthesis and Analysis

Data extraction from each study was conducted by the first author. Structured forms were designed for each of the five main sections of the systematic review: neutrophils, immune complexes and complement, T-cellular immunity, cytokines, and other immunological molecules or factors involved in the pathophysiology of ENL. Data were collected on the setting (study location and country of affiliation of the authors), study design and characteristics of the subjects (ENL case definition, study population included, number of patients with ENL, control subjects, timing of sampling, treatment for ENL and leprosy treatment), study measures and main findings reported by the study authors. A study could include multiple measures and therefore be part of more than one section of the systematic review.

## What is the Role of Neutrophils in ENL?

Neutrophils are the predominant immune cell population in human blood and provide protection through phagocytosis, generation of neutrophil extracellular traps (NETs), and secretion of antimicrobial peptides (18). Recent evidence supports a role for neutrophils in the orchestration of adaptive immunity, engaged with lymphocytes and antigen-presenting cells (APCs) (19).

Neutrophils are considered to be the histological hallmark of ENL (7, 13). The histology of ENL skin lesions shows an intense perivascular infiltrate of neutrophils throughout the dermis and subcutis (7, 13). However, not all ENL lesions are characterized by the presence of neutrophils (12, 20–22) and the timing of biopsies appears crucial in detecting neutrophil infiltration (7, 23). A study of skin biopsies of ENL lesions within 72 h of onset showed a predominance of neutrophils in 30.4% of biopsies. Skin biopsies performed between 9 and 12 days showed neutrophils in 1.6% of specimens and increasing numbers of lymphocytes, plasma cells, and histiocytes (7). Neutrophils may precede the chemotaxis of lymphocytes into ENL lesions, but it is unclear why neutrophils are not always present in the initial stage of ENL.

The study by Lee et al. used DNA microarray and bioinformatic pathway analysis of gene expression profiles in skin biopsies obtained from six patients with ENL compared to seven LL controls (24). They identified 57 functional groups and 17 canonical pathways characteristic of ENL. Their striking finding was the “cell movement” functional pathway composed of 188 genes. From the list of genes of the “cell movement” pathway, 25 were identified to be involved specifically in neutrophil recruitment including the genes for P-selectin, E-selectin, and its ligands (24). Using immunohistochemistry, they showed that E-selectin was expressed in a vascular pattern and at higher levels in ENL skin lesions than in LL, although this was not quantified (24). They described an integrated pathway of TLR2/Fc Receptor activation triggering induction of interleukin (IL)-1β, which together with interferon (IFN)-γ, induced E-selectin expression on endothelial cells and neutrophil migration and adhesion to endothelial cells (24). Interestingly, thalidomide inhibited this neutrophil recruitment pathway (24).

A recent Brazilian study reported that surface CD64 (FcγRI) expression on circulating neutrophils increased significantly during ENL, while BL/LL patients without ENL had lower levels of CD64 (25). In addition, CD64 expression on neutrophils decreased after thalidomide treatment (25). Moreover, the higher levels of CD64 on circulating neutrophils were correlated with disease severity (25). This study demonstrated the potential of CD64 as an early biomarker for ENL and as a marker of severity (25). CD64 (FcγRI) is the high-affinity receptor for monomeric IgG1 and IgG3 (26). While resting neutrophils express low levels of CD64 (26), an increase of neutrophil CD64 surface expression is observed in certain Gram negative bacterial infections (27) and has been associated with the prognosis of disseminated intravascular coagulation during sepsis (28). The authors suggested that CD64 upregulation during ENL could be due to the presence of inflammatory cytokines such as IFN-γ and GM-CSF (29) or certain intracellular components of fragmented *M. leprae* bacilli following treatment with MDT (25). This was further supported by clinical studies showing that although ENL may also occur before initiation of treatment with MDT, the incidence of ENL is higher during treatment with MDT (5, 30).

Studies in the 70s tried to assess the polymorphonuclear leukocyte (PMN) functions in different forms of leprosy and ENL, investigating whether ENL is associated with PMN activation (31, 32). The nitro blue tetrazolium (NBT) test that measures PMN activation was increased in six patients described as LL with leprosy reactions compared with non-reactional leprosy patients (from across the leprosy spectrum) and healthy controls (31). In addition, LL patients with reactions had lower PMN activation when treated with steroids or thalidomide, although this was not significant (31). Another study found the resting NBT levels in different leprosy groups (tuberculoid, lepromatous, and patients with ENL) to be within normal limits (32). However, the sera from patients with ENL produced significantly increased levels of PMN activation as measured by the NBT test when incubated with PMN cells from healthy controls and patients with ENL (32). This finding suggested that sera from ENL patients may lead to activation of neutrophils. However, when cell motility was studied as a marker of PMN activation using random migration, chemotaxis, and chemokinesis, all three were defective in lepromatous patients with or without complicating ENL (32).

Oliveira et al. reported the apoptotic rate of neutrophils to be greatly accelerated in ENL patients compared to BL/LL patients and healthy volunteers (33). Neutrophils isolated from leprosy patients (ENL and BL/LL) released TNF-α and IL-8, after stimulation with lipopolysaccharide (LPS) or *M. leprae* (33). Interestingly, *in vitro* TNF-α production by neutrophils was inhibited by thalidomide at both 3 and 6 h post-stimulation with LPS (33). This supports the role of neutrophils as effector cells actively producing pro-inflammatory cytokines and not only as migratory cells following chemoattractants.

There is little direct evidence of the actual role of neutrophils in ENL, despite the cell being the histological hallmark of ENL. There are multiple histological studies showing the presence of neutrophils in ENL lesions; however, only five studies investigated whether neutrophils actively take part in ENL as effector cells (Table 1). It remains unclear whether the neutrophil initiates ENL or is recruited to the site of the affected skin lesion under the action of chemokines such as IL-8 secreted by other cell types.

**Table 1:** **Studies of neutrophils in ENL**.

Reference; study site(s)	Study population	Timing of screening	MDT status	ENL treatment	Type of samples	Measures	Findings
Goihman-Yahr et al. (31); Venezuela	6 ENL, 32 BL/LL, 6 treated ENL, 9 indeterminate, 11 tuberculoid, 14 HC	ND	ND	Excluded patients on steroids except treated ENL	Peripheral blood neutrophils	Reduction of nitro blue tetrazolium (NBT)	Increased neutrophil activation in ENL
					Serum	Neutrophil response to endotoxin	Lower neutrophil activation after ENL treatment
					Plasma	Effect of adding sera and plasma from ENL to neutrophils of HC	Sera from ENL did not activate neutrophils from HC

Sher et al. (32); South Africa	8 ENL, 17 BT, 11 lepromatous, HC	ND	ND	ENL not receiving steroids or other anti-inflammatory drugs	Peripheral blood neutrophils	PMN leukocyte motility	Defect in random migration, chomotaxis, and chemokinesis in both ENL and lepromatous patients
					Serum	Reduction of nitro blue tetrazolium (NBT)	Reconstitution of PMN leukocytes from HC and ENL with sera from ENL led to increased neutrophil activation

Oliveira et al. (33); Brazil δ	10 BL/LL:6 ENL, 10 HC	ND	On MDT	ND	Peripheral blood neutrophils	Apoptosis	Increased apoptosis in ENL
						DNA fragmentation extracted from neutrophils	Stimulated neutrophils secrete IL-8 and tumor necrosis factor (TNF)-α
						TNF-α and IL-8	

Lee et al. (24); USA ε	6 ENL, 7 LL	ND	ND	ND	Skin	Microarrays and gene expression analysis	Genes involved in neutrophil recruitment identified
						Ability of HUVEC to bind neutrophils from HC	Thalidomide diminished neutrophil binding to HUVECs stimulated with cytokines

Schmitz et al. (25); Brazil ε	62 leprosy: 22 ENL, 16 HC	ENL: before and 7 days after thalidomide	Patients before and after MDT	ENL: before and after thalidomide	Peripheral blood neutrophils	CD64 expression	CD64 upregulated on neutrophils during ENL
							Higher CD64 on neutrophils from severe ENL
							CD64 decreased after thalidomide

*β, also in Table 2; γ, also in Table 3; δ, also in Table 4; ε, also in Table 5*.

*BB, mid-borderline leprosy; BL, borderline lepromatous leprosy; BT, borderline tuberculoid leprosy; ENL, erythema nodosum leprosum; HC, healthy controls; HUVEC, human umbilical vein endothelial cells; ICs, immune complexes; LL, lepromatous leprosy polar; ND, not described; PMN, polymorphonuclear; SLE, systemic lupus erythematosus; TB, tuberculosis; TT, tuberculoid leprosy polar*.

## What is the Role of Immune Complexes in ENL?

An IC or antigen-antibody complex is the result of binding of one or more antibody molecules with one or more antigen molecules (34). The ability of ICs to activate the complement system and to interact with a number of cells determines their biological properties (35). ICs activate complement pathways that opsonize or coat antigen–antibody complexes with large numbers of C3 molecules (36). Opsonization facilitates the clearance of ICs by the macrophage system (36). By maintaining complexes in solution, the complement allows clearance of ICs from their site of formation, minimizing local inflammatory consequences (36).

It was hypothesized that ENL is an IC-mediated disorder because it has some clinical features in common with the Arthus reaction, a type III hypersensitivity reaction that involves the deposition of ICs mainly in the vascular walls, serosa, and glomeruli and is characterized histologically by vasculitis with a polymorphonuclear cell infiltrate (37). The multisystem involvement of ENL resembling autoimmune diseases associated with ICs such as systemic lupus erythematosus (SLE), also lends credence to this theory.

Multiple studies have been performed investigating ICs in ENL. The widely cited study of Wemambu et al included 17 patients with ENL and six uncomplicated LL controls (37). Direct immunofluorescence demonstrated granular deposits of immunoglobulin and complement in a perivascular distribution in association with a polymorph infiltrate in the dermis of 10 out of 17 ENL lesions but not in any lesions of uncomplicated LL (37). However, such deposition is not conclusive evidence of ICs. The presence of soluble mycobacterial antigen was seen in ICs in only 3 out of 17 ENL lesions (37). The authors hypothesized that ENL results from the deposition of ICs in and around venules of the connective tissue septa of subcutaneous fat (37). The study was repeated using 38 patients with ENL and 13 LL controls and demonstrated the presence of immunoglobulin, complement, and mycobacterial antigen in less than half of the skin biopsies from patients with ENL and none of the LL control biopsies (22). Non-specific granular deposits of IgG were demonstrated along the collagen and elastic fibers in the dermis of all 25 patients with ENL in another study, not in any of the 10 LL patient controls (38). However, the deposits were not consistently seen in and around the blood vessels (38). Later studies in ENL suggest that these ICs are extravascular and hence ENL differs from the Arthus reaction (39, 40). These studies taken together provide evidence of an association of ICs and ENL but they do not necessarily support that ICs are the trigger leading to ENL.

Circulating ICs have been demonstrated in patients across the leprosy spectrum (41). The level of circulating ICs in the sera of leprosy patients have been measured in many studies using different immunological techniques (42–54) of which the most commonly used are C1q immunoassays (42, 43, 51). This highlights the fact that the use of different immunoassays to detect circulating ICs in studies may explain the contradictory results. The first study measuring ICs in sera of leprosy patients performed C1q immunoassays in samples from LL patients, tuberculoid leprosy patients, and healthy volunteers and showed that more than 70% of LL patients had demonstrable ICs (43). A subsequent study demonstrated increased occurrence of ICs in both the sera of ENL patients (80%) and uncomplicated LL patients (82%), indicating that the presence of circulating ICs is not a characteristic feature of ENL *per se* (46). Wager et al. analyzed sera from 135 leprosy patients using the platelet aggregation test (PAT) which had been previously suggested to be a sensitive detector of IgG complexes in other immunological and infectious diseases (55, 56) and concluded that PAT is a sensitive detector of IgG complexes peculiar to LL (44). No ICs were detected in the sera of leprosy patients using the C1q immunoassay (44).

Specific mycobacterial antigens (41) or antibodies against *M. leprae* antigens (50, 57) have been identified in the ICs derived from sera of lepromatous patients with or without ENL. Rojas et al. precipitated ICs from sera and detected antibodies against phenolic glycolipid-1 (PGL-1) (50) and major cytosolic protein of *M. leprae* (MCP-I). The finding that ICs are composed of anti-PGL-I and anti-MCP-I antibodies supports the concept that ENL is an IC-mediated disorder (50). However, the composition of circulating ICs of leprosy controls (combined BT and BL/LL) also showed high levels of anti-PGL-I antibodies (50) again suggesting that ICs are not specific to ENL.

Dupnik et al. used DNA microarrays to examine gene expression in PBMC isolated from patients with ENL and matched leprosy controls (58). Several components of the classical complement pathway showed increased expression in PBMC from patients with ENL: C1qA, B, and C and the complement receptors C3AR1 and C5AR1 (58). Increased intensity of fluorescent staining for C1q in skin lesions of ENL compared to BT and BL/LL controls was demonstrated (58). The finding of increased C1q deposition in the skin of ENL does not necessarily mean IC deposition has occurred (35). However, these data do support activation of the classical complement pathway in ENL, which may result from antigen–antibody formation.

Earlier studies in leprosy looked at the role of free complement in the sera of lepromatous patients (59). The serum C3 levels were decreased in patients with ENL, whereas they were elevated in LL controls (60). The low levels of C3 supported the concept that ENL is mediated by an antigen-antibody reaction and may be due to its utilization during the course of such antigen-antibody reactions. Similar decreased serum complement levels have been reported in other IC disorders such as acute glomerulonephritis (61–63) and acute systemic lupus erythematosus (SLE) (64, 65). It has been suggested that ENL is characterized by complement hypercatabolism because the level of the C3 breakdown product C3d in the sera was increased in 70% of the patients with ENL but in only 18% of patients with uncomplicated LL (46).

In other IC-associated diseases such as SLE, systemic vasculitides, and nephritis, defective complement-mediated solubilization of immune precipitates have been observed (48, 49, 66). Similarly, leprosy patients with ENL were shown to have markedly reduced solubilization levels that remained low for 3 months, whereas the C3d and circulating IC levels returned to baseline levels (48). Circulating ICs isolated from sera across the leprosy spectrum as PEG precipitates were shown to be efficient activators of the alternative complement pathway. In addition, PEG precipitates from BL/LL leprosy patients including those with ENL were shown to activate the classical complement pathway as well (52).

A Brazilian study of 46 patients with ENL investigated the association between the MHC class III complement proteins C2, BF, C4A, and C4B and leprosy (67). All patients who were homozygous for the silent C4B allele (C4B*Q0) and thus C4B-deficient had ENL (67). Increased frequency of ENL was also associated with those who were hemizygous for the C4B*Q0 allele. The relative risk of patients suffering from ENL carrying the C4B*Q0 allele was 5.3 compared with LL patients without C4B*Q0 (67). Interestingly, their findings suggested that C4B deficiency could play an important role in the abnormal immune response to *M. leprae* and to the lack of IC clearance, leading to ENL reactions (67). Hemizygous C4 deficiencies are associated with immune complex diseases such as SLE (68).

There is lack of evidence to support a causative role of ICs in ENL, which requires the deposition of ICs in tissues, the presence of bacterial antigens in these ICs, and the interaction of the ICs with the complement cascade and with phagocytic cells (35). Although there are 28 studies investigating the presence of ICs in the skin or circulating ICs in the sera of patients with ENL (Table 2), their role remains uncertain. It is unclear whether they are involved in the pathogenesis of ENL or simply an epiphenomenon.

**Table 2:** **Human studies on ENL investigating immune complexes and complement**.

Reference; study site(s)	Study population	Timing of screening	MDT status	ENL treatment	Type of samples	Measures	Findings
de Azevedo and de Melo (59); Brazil	37 lepromatous, 33 tuberculoid, 18 “lepra reaction”	ND	ND	ND	Serum	Complement unit (K)	Reduced complement activity in the reactional group
						Angular inclination (1/n)	

Wemambu et al. (37); United Kingdom and Malaysia	17 ENL, 6 lepromatous	ND	ND	ND	Skin	Immunoglobulin	Perivascular deposits of immunoglobulin and complement
					Serum	Complement	Mycobacterial antigen in some ENL skin lesions

Waters et al. (22); United Kingdom and Malaysia ε	38 lepromatous with ENL, 13 lepromatous	ND	ND	ND	Skin	Immunoglobulin and complement	Immunoglobulin and complement perivascular in some ENL skin lesions
					Serum	Detection of mycobacterial antigen in the ICs	Mycobacterial antigen present in ICs

Gelber et al. (69); Taiwan and USA	15 LL with ENL, 47 BT-LL	3 or more specimens time-span up to 4 months in BT and LL/ENL and up to 6 months to LL without ENL	ND	ND	Serum	C1q precipitin activity	Association of C1q precipitin activity with ENL
						Complement levels C3	
						Cryoglobulins	

Bjorvatn et al. (46); Ethiopia and Switzerland	13 ENL, 7 LL, 6 tuberculoid, pulmonary TB, 30 HC	ND	All on dapsone or clofazimine	ENL patients received treatment for ENL	Serum	ICs with ^125^I-C1q binding assay	ICs increased in ENL and LL but also in tuberculoid leprosy
						Complement	Increased C3d level in most patients with ENL

Tung et al. (51); Ethiopia	22 BL/LL with ENL, 23TT-LL, 17 SLE	ND	19/23 non-ENL on dapsone	Untreated ENL	Serum	C1q ICs	Circulating ICs in 67% of leprosy by C1q test
						Raji test ICs	Only 7% of this 67% showed ICs by the Raji test

Anthony et al. (60); India ε	25 LL with ENL, 10 LL without ENL	Active ENL lesions at the time of the biopsy	ND	ND	Skin	Immunoglobulin deposits in skin	Immunoglobulin deposits in ENL skin but not in LL
							Decreased serum complement in ENL
					Serum	Complement in sera	Elevated levels in LL

Wager et al. (44); Finland, Brazil, and Ethiopia	11 ENL, 112 leprosy, 61 LL, 7 tuberculoid, 28 SLE, 42 RA, 374 HC	ND	ND	ND	Serum	ICs with Platelet aggregation test (PAT)	Higher PAT titers toward the lepromatous end of the spectrum
						Other sero-immunological parameters	No significant differences for ENL patients

Izumi et al. (70); Japan γ	12 ENL, 49 active lepromatous, 24 inactive lepromatous, 7 borderline, 6 tuberculoid, 9 HC	ND	ND	ND	Serum	C4, C3c, C3 activator	C3 activator and C3c concentrations higher in ENL compared with active lepromatous

Harikrishan et al. (71); India ε	20 active LL, 15 ENL active and subsided, 20 HC	ENL: during the active and the subsided phase	ND	ND	Serum	Complement factor C3	Increased levels of C3 in LL and ENL
							Decrease in C3 during the “subsided phase” of reaction

Saha et al. (72); India	20 ENL, 15 HC	Initial sample first visit, subsequent on ENL clinical remission 4 weeks later	ND	Second sample: on antireactional treatment	Serum	Complement C1q, C3, C4	C3 level decreased during ENL, while increased after remission C3d increased during ENL, and remained elevated after clinical remission in most patients
							No significant difference in C1q or C4 during ENL

Valentijn et al. (54); Netherlands and Surinam	70 leprosy throughout the whole spectrum, 11 HC	ND	ND	ENL patients possibly on thalidomide treatment?	Serum	ICs	Elevated C3d significantly associated with ENL
						Complement C1q, C3, C4	

Mshana et al. (73); Ethiopia	26 ENL, 20 BL/LL	Skin biopsies of ENL less than 12 h old	ND	ND	Skin	Immunoglobulin deposits Complement deposits	No ICs around blood vessels in ENL lesions IC formation common feature in LL
						Mycobacterial antigens	Absence of immunoglobulin or C3 deposits in early ENL
							Extracellular antigens not seen

Ridley and Ridley (39); Malaysia, PNG, Ethiopia, and UK	20 ENL, 10 non-reactional leprosy	ND	ND	ND	Skin	Immunoglobulins IgG, IgM, IgA, IgE Complement C3, C4, C1q, C3d	ENL lesions had disintegration of macrophages and release of bacterial antigen combined first with IgM, later with IgG, present together with complement components of the classical pathway
							ICs were both extracellular and in neutrophils and macrophages
							ICs were extravascular

Ramanathan et al. (74); India	10 BT, 10 LL, 10 BT reactional, 30 LL reactional	ND	All patients on dapsone	Sampling before antireactional treatment	Serum	C3 and C4	Increased C3d in both BT reactional (T1R) and LL reactional
						ICs Isolated ICs for IgG, IgA, IgM, C3, C4 and antimycobacterial antibody	Circulating ICs in all reactional patients No antimycobacterial antibody in ICs from LL reactional patients

Saha et al. (75); India	20 ENL, 15 HC	Before and 4 weeks after starting treatment for ENL	ND	Second sample: on antireactional treatment	Serum	Quantitative analysis of composition of PEG precipitates (immunoglobulins, complement components, autoantibodies and acute phase proteins) and anticomplementary activity of PEG precipitates	Anticomplementary activity of PEG precipitates more in the lepromatous than in normal sera, independent of the presence of ENL

Ramanathan et al. (48); India	32 LL in reaction, 10 BT, 10 BT in reaction, 10 LL uncomplicated, 15 HC	ND	BT and LL without reaction treatment for at least 2 years; all patients were on dapsone	ND	Serum	ICs by fluid phase ^125^I-labeled conglutinin binding assay; serum C3d Complement-mediated solubilization of immune precipitates	Reduced solubilization of *in vitro* formed immune precipitates by the sera of ENL patients C3d, ICs, and solubilization levels correlated with the clinical course of reaction ICs and C3d decline after clinical subsidence of ENL

Sehgal et al. (76); India ε	21 T1R or ENL	ND	ND	ND	Serum	Complement C3	Lower level of C3 during ENL

Chakrabarty et al. (77); India	27 BB-BL-LL: 7 ENL, 4 T1R	Initial blood collected at the onset of reaction and subsequent 4 weeks after ENL remission	Patients on MDT	Second blood sample on antireactional treatment	Serum	Solubilization of preformed ICs (^125^I-BSA-anti-BSA complexes)	The mean solubilizing capacity of the reaction patients’ sera during the reaction was not significantly different from LL without ENL
							After clinical remission of the reaction, most patients showed no increase in the ICs solubilization

Rao and Rao (78); India ε	44 ENL, 39 BL/LL, 22 post-ENL	ENL: before starting treatment with anti-inflammatory drugs/steroids	20 BL/LL untreated and 19 BL/LL treated with dapsone less than a year	Untreated first sample and second post-ENL sample after discontinuation of antireactional treatment	Serum	C3 and C4 levels IgG, IgA, IgM, C3, and C4 levels in the ICs ICs by PEG method	C3 and C4 levels were not significantly different in ENL compared to BL/LL and post-ENL C3 and C4 levels in the ICs reduced insignificantly in ENL than BL/LL and post-ENL IgG, IgA, and IgM in ICs showed no significant differences from LL to ENL and post-ENL
		Post-ENL: ensuring that the patient had not taken anti-inflammatory drugs/steroids for at least 3 or 7 days					

Sehgal et al. (79); India	17 ENL	Before antireactional treatment and 1 week after the clinical subsidence of ENL	On MDT	First sample untreated and second sample on prednisolone in 10 patients	Serum	Complement components: classic pathway: C1q and C4 Alternative pathway: C3, C3d, Factor B	No significant change in classical pathway in ENL reaction C3 elevated, C3d decreased and increase of Factor B after ENL

Jayapal 1989 (47); India	37 leprosy: 9 ENL, 6 bacterial endocarditis, syphilis, SLE, HC	ND	All leprosy patients on dapsone	ENL patients on clofazimine, prednisolone, antihistamine and chloroquine	Serum	ICs with PEG method	ICs higher in ENL than in LL

Sehgal et al. (81); India	18 T1R, 17 ENL, non-reactional controls	During and after reaction	On MDT	ND	Serum	Complement components: classic pathway: C1q and C4 Alternative pathway: C3, C3d, Factor B	Classic pathway: no significant change in C1q and C4 during ENL Alternative pathway: increase in C3d during ENL; decrease of C3 during ENL; reduction of Factor B during ENL; elevation of C3 and Factor B after ENL

Tyagi et al. (52); India	20 BL/LL with ENL, 20 TT/BT, 20 BT with reaction, 20 BL/LL; 15 HC	ND	ND	ND	Serum	ICs by PEG precipitation Mycobacterial ICs in PEG precipitates; CH50 assay and AH50 assay (complement consumption)	PEG ICs from BL/LL and ENL higher IgG and IgM antimycobacterial antibodies than TT/BT, BT reactional (T1R) and HC No significant functional differences between the PEG ICs from reactional and non-reactional leprosy

Ramanathan et al. (49); India ε	26 BL/LL: 11 ENL, 24 HC	Before initiation of treatment and 2-monthly intervals	Untreated and then on MDT	Treated but after sampling	Serum	ICs by PEG method	High levels of ICs in both LL and ENL
						C3d	Lower levels of complement-induced IC solubilization in ENL
						Complement-induced IC solubilization	Highest levels of ICs and C3d at the time of ENL

Scollard et al. (82); Thailand ε	4 cured leprosy, 10 non-reactional leprosy BT/BL/LL, 8 ENL patients (5 LL/3 BL), 3 T1R, 4 HC	ND	ND	ND	Blisters induced over representative skin lesion	ICs	ICs in ENL similar to that of active leprosy (either lepromatous or tuberculoid) Higher ICs in blisters than in matching sera
					Serum		

Rojas et al (50); Brazil ε	19 ENL, 10 BL/LL, 13 family contacts; 15 healthy non-contacts	ND	Both untreated and patients on MDT for 1–72 months	ND	Serum	ICs; anti-PGL-I IgM in IC precipitated from sera; anti-10-kDa hsp IgG in IC precipitated from sera	ENL highest levels of ICs compared with all other groups IgM anti-PGL-I and IgG anti-MCP-1 heat shock protein antibodies constituents of ICs in ENL

Dupnik et al. (58); Brazil δ, ε	11 ENL, 11 T1R, 19 non-reactional leprosy, additional 6 ENL, 11T1R, 11 HC	ND	3 ENL pre-treatment, 2 ENL on treatment and 6 ENL post-treatment; Leprosy controls matched for stage of treatment	Excluded patients who had received corticosteroids within 7 days or thalidomide within 28 days of enrollment	PBMC	Microarray and qPCR for transcriptional profile of PBMC; IHC for C1q in skin lesions	Complement and coagulation pathway common in ENL and T1R
					Skin		Transcripts uniquely increased in ENL included complement receptors C3AR1 and C5AR1
							C1q staining higher in both ENL and T1R compared with non-reactional leprosy

*α, also in Table 1; γ, also in Table 3; δ, also in Table 4; ε, also in Table 5*.

*BB, mid-borderline leprosy; BL, borderline lepromatous leprosy; BT, borderline tuberculoid leprosy; ENL, erythema nodosum leprosum; HC, healthy controls; ICs, immune complexes; LL, lepromatous leprosy polar; ND, not described; PEG, polyethylene glycol; PNG, Papua New Guinea; RA, rheumatoid arthritis; SLE, systemic lupus erythematosus; TB, tuberculosis; TT, tuberculoid leprosy polar; WHO, World Health Organization*.

## What is the Role of T-Cells in ENL?

T-lymphocytes are part of the adaptive immune response which help to eliminate bacterial, viral, parasitic infections or malignant cells. The antigen specificity of the T-cell is based on recognition through the T-cell receptor (TCR) of unique antigenic peptides presented by major histocompatibility complex (MHC)-molecules on APCs: B cells, macrophages, and dendritic cells. There are two major T-cell lineages, defined by the presence of two surface co-receptor molecules, namely, CD4 and CD8. CD4^+^ cells when they are activated produce cytokines as effector T helper cells, whereas CD8^+^ lymphocytes form effector cytotoxic T lymphocytes (CTL). Furthermore, activated CD4^+^ T helper cells can be subdivided into Th1, Th2, Th17, and T regulatory (Treg) subsets based on the production of signature cytokines (83).

Early studies investigating T-cell biology in the pathophysiology of ENL reported that ENL patients had higher T-cell numbers in peripheral blood than uncomplicated LL patients, although both LL and ENL patients had a significantly lower percentage and absolute number of T-cells compared to healthy controls (84). In addition, the high numbers of T-cells observed during ENL remained high post-ENL treatment compared to the LL controls (85).

Patients with ENL had increased CD4^+^ T cell numbers and a simultaneous decrease in CD8^+^ T cell numbers and an increased CD4^+^/CD8^+^ ratio in the blood compared to LL controls (86, 87), while ENL patients had decreased CD4^+^/CD8^+^ ratio after successful treatment. This ratio increased in those patients who had an ENL recurrence (87). An increased CD4^+^/CD8^+^ ratio in ENL patients was reported by several subsequent studies (87–92). In acute SLE, it has been suggested that the failure of CD8^+^ T-cell activity could lead to increased IgG production and to the subsequent formation of ICs (93). However, there are studies in ENL reporting a decreased CD4^+^/CD8^+^ ratio compared to non-reactional LL controls (94) or a similar ratio (95, 96).

The first immunohistological studies of T-cell subsets in skin lesions included small numbers of ENL patients and assessed the percentage and ratio of CD4^+^ and CD8^+^ T cells by comparing them to non-ENL lepromatous specimens (89, 91, 97–103). ENL skin lesions, like peripheral blood, were characterized by an increased CD4^+^/CD8^+^ ratio in all but one of these studies (89, 91, 97–100, 102, 103).

CD4^+^ T cells differentiate according to the microenvironment into Th1, Th2 cells, or subsets of Th17 and Treg (104). Recent studies have reported the frequency of the newly described Th17 and Treg subsets in leprosy (105, 106). Using flow cytometry in ENL, the absolute numbers and proportion of Tregs were shown to be significantly lower during ENL although FoxP3 expression, a marker they used to define Tregs, was higher (107). Tregs suppress or downregulate induction and proliferation of effector T cells (108). Therefore, the observation of lower numbers of Tregs in ENL could account for the relatively higher proportion of T cells previously described in multiple ENL studies. Two more publications from the same group addressed the frequency of Tregs in ENL, defined as CD4^+^CD25^high^ FoxP3^+^ cells and reported the ratio of Treg/Teffector cells to be low in ENL (109, 110). These results should be interpreted with caution since dichotomizing cells into CD25^high^ and CD25^low^ to identify Tregs is highly subjective. There is no consensus on the thresholds of CD25 expression to delineate Tregs within the CD25^high^ population (111). Variations in FoxP3 expression within the CD25^high^ population have been observed even in healthy individuals (112).

A recent study that used flow cytometry described a significant reduction in percentage of CD4^+^CD25^+^FoxP3^+^ Treg cells and mean fluorescence intensity of FoxP3 in PBMC in patients with ENL compared to LL controls (113). The observed reduction of Tregs in ENL patients could lower the inhibitory effects on effector T cells and therefore lead to enhanced Th17 activity, tipping the balance toward inflammation, as previously described in other conditions such as tuberculous pleural effusion (114). Interestingly, an increase of FoxP3 mRNA expression by PBMC in ENL patients compared to LL controls has also been reported (113). The conflicting results for FoxP3 could be due to variation in the flow cytometry gating or the fact that FoxP3 mRNA may not be translated to functional FoxP3. A previous study measured the expression of Foxp3 by qPCR in skin biopsies and PBMC of five patients with ENL and detected Foxp3 in all skin and PBMC samples. An upward trend of Foxp3 in PBMC was described during the first 21 days of thalidomide treatment (115). The authors suggested that thalidomide may boost Tregs by T-cell costimulation *via* CD28 and therefore augment the IL-2-dependent number and/or function of Tregs (115). However, the changes in Foxp3 expression did not reach statistical significance, while no IL-2 mRNA was detected in any samples (115). Another study addressed FoxP3 expression by immunohistochemistry in skin but there was no difference in patients with ENL compared to non-reactional leprosy controls across the spectrum (116). Recent research suggests that Tregs constitute a stable cell lineage whose committed state in a changing environment is ensured by DNA demethylation of the Foxp3 locus irrespective of ongoing Foxp3 expression (117). Further investigation is needed to better define the role of Tregs in the pathogenesis of ENL.

Patients with ENL do not exhibit the phenomenon of “anergy” of cell-mediated immune response observed in untreated LL patients (118). Patients with ENL had elevated mean proliferative responses to several mitogens compared to uncomplicated LL patients (86, 87), while an enhancement in T-cell-related functions during the acute phase of an ENL reaction has also been described (94).

The interpretation of the role of T cell subsets in ENL is hampered by small sample sizes and methodological issues. 63.6% of the 44 studies investigating the role of T-cells in ENL (Table 3) are cross-sectional and lack serial sampling before and after treatment for ENL. However, it appears that T cell subsets do play an important role in ENL because multiple studies report an increased CD4^+^/CD8^+^ ratio in ENL patients in both skin and peripheral blood.

**Table 3:** **Human studies on ENL investigating T-cell biology**.

Reference; study site(s)	Study population	Timing of sampling	MDT status	ENL treatment status	Type of samples	Measures	Findings
Lim et al. (84); USA and Korea (mixed ethnic background)	7 LL ENL, 20 active LL, 9 inactive LL, 4 BB, 3 indeterminate leprosy	ND	All patients treated with Dapsone or Clofazimine or Rifampicin for varying durations	5 had received various doses of steroids and 3 were treated with steroids at the time of the study	Blood	T lymphocyte numbers by the rosette assay	ENL showed T-lymphocyte numbers significantly higher than LL
							LL had lower T-lymphocyte numbers than HC

Anders et al. (119); Papua New Guinea	31 leprosy: 13 BL/LL with amyloidosis (11/13 frequent ENL), 9 BL/LL ENL without amyloidosis, 9 BL/LL with few or no ENL episodes	ND	Approximately half patients on clofazimine and other half on dapsone	2 ENL at testing: 1 steroids and 1 stibophen	Blood	Lympohocyte transformation tests	Patients with a history of frequent ENL had greater cell-mediated responses to PHA than patients without ENL

Izumi et al. (70); Japan β	12 ENL, 49 active lepromatous, 24 inactive lepromatous, 7 borderline, 6 tuberculoid, 9 HC	ND	ND	ND	PBMC	Percentage and number of Tμ (T cells with Fc receptor for IgG) and Tγ (T cells with Fc receptor for IgM)	No significant differences between different clinical groups

Bach et al. (86); France (multiple ethnic groups)	9 BL/LL with no recent history of ENL, 9 BL/LL suffered from ENL less than 2 months prior to the investigation, 13 BT/TT, HC	ND	Some untreated and others on MDT	Certain ENL on antireactional treatment	Blood	T cell subsets; Proliferative responses to mitogens	Increased %age of helper T cells in ENL
							Decreased %age of suppressor T cells in ENL
							Elevated proliferative responses to mitogens in ENL
						Con A-induced suppressive activity	Most ENL decrease of suppressive index, whereas none of the LL or TT patients had a diminished suppressive activity

Dubey et al. (120); India	41 untreated cases of leprosy, 64 TT and LL taking antileprosy treatment, reactional (8 ENL and 10T1Rs), 11/41 follow-up from untreated leprosy patients	ND	64 cases on antileprosy treatment	Untreated cases of ENL?	Blood	Lymphocytic culture: percentage of Blast transformation	Blast percentage in ENL slightly higher than T1R

Mshana et al. (90); Ethiopia	21 BL/LL, 10 BT, 5 ENL	ND	All patients received MDT but unclear whether sampled prior to MDT	No patient on thalidomide	Blood	Lymphoproliferative responses to PPD or PHA T-cell subsets	Higher responses to PPD or PHA in ENL Decreased number of suppressor cells prior to ENL, which increased with clinical recovery from ENL

Mshana et al. (88); Ethiopia	69 leprosy patients: 26 ENL, 13 HC	Untreated samples	Untreated samples	Untreated samples	Blood	T lymphocyte subpopulations; lymphoproliferation using *M. leprae*, PHA and PPD	ENL patients had decrease in suppressor cells and an increase of CD4^+^/CD8^+^ ratio compared to LL ENL had higher responses to both PHA and PPD BL/LL patients with or without ENL lower proliferative responses to *M. leprae* than BT patients and HC

Wallach et al. (87); France (samples from multiple ethnic groups)	9 recent ENL, 6 bacteriologically positive patients of which 1 ENL more than 5 years ago, 9 treated leprosy patients of which 3 had ENL	Described in detail each patient duration of disease	All treatment described in detail	Some on antireactional treatment	Blood	T cell subsets; Lymphocyte transformation tests: proliferative responses to mitogens	ENL patients have elevated Helper/Suppressor ratio
							Mean proliferative responses elevated in ENL

Bach et al. (121); France	8 treated lepromatous without recent ENL with BI < 1 +, 6 lepromatous with BI > 2+ (untreated or suffering a relapse, without recent ENL reaction), 12 lepromatous who underwent at least one ENL episode, 13 tuberculoid, 41 HC	ND	ND	ND	PBMC	T-cell subsets; Proliferative response to *M. leprae* and PPD of isolated T-cell subsets	ENL decreased CD8^+^ T cell percentages and increased CD4^+^/CD8^+^ ratios T-cell subset percentages returned to normal either when the bacterial load was reduced by treatment or when the ENL reaction resolved ENL episodes associated with improvement of T-cell unresponsiveness to various antigens or mitogens

Modlin et al. (97); USA	15 non-reactional leprosy BT/BB/BL/LL, 17 reactional (6 T1R, 9 ENL, 2 Lucio’s reaction)	ND	Results did not differ between treated and untreated subjects	3 ENL had no therapy	Skin	T lymphocyte subsets	The helper/suppressor ratio in ENL was significantly higher than in non-reactional lepromatous disease

Modlin et al. (98); USA	14 leprosy patients (4 tuberculoid, 2 borderline in T1R, 1 BL, 7 lepromatous of which 5 ENL), 8 HC	ND	6 treated patients	ND	Skin	T lymphocyte subsets	ENL lesions showed 2:1 predominance of helper cells whereas in the lesions without ENL the helper: suppressor ratio was 1:1 smaller

Sasiain et al. (122); Argentina	16 ENL, 12 HC	First blood sample ND; 9 ENL 20-30 days after stopping thalidomide	All patients on MDT	Thalidomide in patients with ENL	PBMC	ConA-induced suppressor response	Suppressor T-cell function was reduced during ENL and after ENL than HC

Narayanan et al. (89); India ε	7 LL ENL, 6 BT T1R, 5BL T1R, 18 BT-LL	ND	ND	ND	Skin	T cell phenotypes	Lesions of ENL showed increase in T cells with a predominance of the helper/inducer subset; CD4^+^/CD8^+^ ratio was higher in ENL and T1R than non-reactional lesions

Rea et al. (96); USA ε	19 ENL, 24 LL non-reactional with treatment, 12 LL non-reactional no treatment, 18 LL with long-term treatment, 4 LL with Lucio’s, 13 BL, 13 T1R, 18 Tuberculoid, 13 Tuberculoid with long-term treatment	ND	Some patients on MDT	ENL before receiving thalidomide	PBMC	T cell subsets	Active LL patients have lymphopenia, a proportionate reduction in the numbers of each of the three T cell subsets
							Insignificant changes in T cell subsets expressed as percentages and in the helper: suppressor ratio

Laal et al. (94); India ε	15 ENL, 13 LL	During active ENL and 1 week to 4 months after stopping treatment	On MDT	First sample before initiation of antireactional treatment	Blood	Leukocyte migration inhibition test	ENL significant inhibition of antigen-induced leukocyte migration
						Lymphoproliferation	Lymphoproliferation enhanced during the acute phase of ENL
				Second sample 1 week to 4 months after stopping treatment		Suppressor cell activity; T cell subsets	Enhanced antigen-stimulated suppression of mitogen responses in ENL
							Leukocyte migration inhibition, lymphoproliferation, and suppressor cell activity were reduced in post-ENL to the unresponsive state seen in stable LL
							Lower CD4^+^/CD8^+^ ratio in ENL compared to LL

Modlin et al. (99); USA	12 ENL and 10 non-reactional leprosy; 19 ENL blood samples	ND	ENL biopsies: 8/12 treated with dapsone; ENL blood: 15/19 treated	Some ENL were treated	Blood	T lymphocyte subsets	ENL tissue more cells of the helper-inducer phenotype and fewer of the suppressor-cytotoxic phenotype, as compared with non-reactional LL
					Skin		No correlation between tissue and blood helper-suppressor ratios

Wallach et al. (91); France	ND	ND	ND	ND	Blood	T cell helper-suppressor (HS) ratio	HS ratio higher in ENL lesions and blood than non-ENL leprosy controls
					Skin		

Modlin et al. (100); USA	Biopsies: 25 ENL, 23 tuberculoid, 23 non-reactional lepromatous;	ND	Some patients received treatment	Some patients on treatment?	Blood	Skin: number of T cells, T cell subsets; Blood: lepromin-induced suppression of the Con A stimulation	Increases in both CD4^+^/CD8^+^ ratio and the number of IL2-positive cells in ENL Suppressor activity decreased significantly in ENL Suppressor activity returned to normal after ENL subsided
	Blood: 18 ENL						
					Skin		

Rao and Rao (123); India ε	44 ENL, 39 BL/LL, 22 post-ENL	ENL patients before starting ENL treatment, post-ENL after patient had not taken anti-inflammatory/steroids for at least 3 and 7 days	From 39 non-reactional cases: 20 untreated and 19 with dapsone for less than a year	Before starting treatment for ENL with steroids or anti-inflammatory drugs, post-ENL: ensuring that the patient had not taken anti-inflammatory drugs or steroids for at least 3 and 7 days, respectively	Blood	Sub-population of T cells with receptors for Fc portion of IgG (Tr) and Fc portion of IgM (Tμ)	Tμ/Tr ratio higher in ENL than lepromatous and post-ENL patients

Rao and Rao (85); India	77 leprosy: 44 ENL	ENL: before starting anti-ENL treatment, post-ENL: After patient had noe taken anti-inflammatory drugs or steroids for at least 3 and 7 days	19 patients treated with dapsone for less than 1 year	Before starting treatment for ENL with anti-inflammatory drugs or steroids	Blood	Leykocyte migration inhibition test (LMIT)	No significant difference in mean migratory index to PHA, PPD, sonicate *M. leprae*
						Enumeration of early and total T lymphocytes	Whole *M. leprae* increased response in ENL compared to LL Lower migratory indices to whole *M. leprae* in post-ENL than LL
							%age of early T lymphocytes increased in ENL compared to LL
							%age of early T lymphocytes remained high in post-ENL compared to LL
							Cell-mediated immune responses enhanced during ENL and return to LL levels once the episode is over

Shen et al. (101); USA	10 ENL, 8TT/BT, 10 BL/LL, 10 T1R	ND	ND	ND	Skin	CD3, CD4, CD8 and Ta1 (memory) positive cells	CD3, CD4 and CD8 showed percentages of positive cells in lesions similar between patient groups
					PBMC		No significant difference in%age of memory T-cells in ENL compared to LL

Bottasso et al. (124); Argentina	8 LL/ENL, 17 LL, 9 TT, 11 HC	ND	Patients on MDT	Patients with ENL were not on thalidomide treatment but unknown whether they were on steroids	Blood	T-Lymphocytes count absolute and relative; Lymphocyte functional assay: capacity of rosetta formation	Active LL showed a decrease in T-lymhocytes
							ENL showed a restoration of the levels of T-lymphocytes

Rasheed et al. (125); Zambia and Pakistan	167 leprosy of which 21 LL/ENL, 12 BL/T1R, 24 BT/T1R, 46 endemic HC	ND	ND	ND	Serum Lymphocytes	Lymphocytotoxic activity	Lymphocytotoxic activity scores were significantly raised in patients with reactions

Sasiain et al. (126); Argentina	53 leprosy patients TT/BT/BB/BL/LL and 9 LL/ENL, 23 HC	ND	Received MDT	Thalidomide for ENL	PBMC	Proportion of CD8^+^ cells	Proportion of CD8^+^ cells was low in LL patients and tended to normalize during ENL episodes
						*M. leprae*-induced suppression of T-cell proliferation; Induction of IL-2R by culture with *M. leprae*	
						PHA- and ConA-induced proliferation	

Bhoopat et al. (127); Thailand ε	57 ENL (19 acute/38 chronic), 61 active LL, 33 cured leprosy	26 BL/35 LL newly diagnosed and untreated	ND	If corticosteroid and/or thalidomide was initiated before or during the study, precise timing of medication was recorded with respect to the time of collection of laboratory specimens	Blisters induced over a representative skin lesion	T cell subsets *in situ*	The lesions of chronic ENL showed a decreased number of CD8^+^ cells and increased helper/suppressor ratio compared to those in acute ENL and non-reactional leprosy; Systemic administration of corticosteroids caused a reduction in the CD4^+^ cell population but did not change CD8^+^ cell population

Rea and Modlin (102); USA δ	ND	ND	ND	ND	Skin	T-cell phenotypes: CD4^+^ versus CD8^+^ cells, γ/δ and α/β receptor-bearing lymphocytes, T-memory and T-naïve cells	ENL lesions predominance of CD4^+^ cells similar to those in tuberculoid (TT/BT?) and T1R
							LL patients showed an excess of CD8^+^cells

Tyagi et al. (53); India	4 TT/BT, 5 BL/LL, 4 ENL	ND	ND	ND	Blood	Effect of isolated circulating ICs from BL/LL or ENL patients to lymphocyte transformation test on T cells of HC	PEG precipitates isolated from BL/LL or ENL subjects had a significant suppressive effect on lymphocyte proliferation in HC

Foss et al. (128); Brazil δ	28 lepromatous: 11 ENL, 23 tuberculoid, 19 HC	ND	lepromatous patients 86% treated with dapsone	11 ENL at time of blood collection no immunosuppressive drug	Blood	T lymphocyte response to concanavalin A	Marked reduction on concanavalin A-induced lymphoproliferation in patients with ENL

Santos et al. (129); Brazil ε	59 LL/BL, 10 ENL, 4 T1R, 4 post-reactional	ND	On MDT	No specific treatment for reactions before blood collection	PBMC	Lymphocyte proliferation after ConA and *M. leprae*	T1R showed greater lymphocyte proliferation compared to all other groups

de la Barrera et al. (130); Argentina	7 TT/BT, 20 BL/LL of which 3 ENL	ND	All patients on MDT	ND	PBMC	T-cell cytotoxic activity induced by *M. leprae* and *M.tb* heat shock protein (HSP)	*M. leprae* hsp65 induced cytotoxic responses only in those MB patients undergoing ENL

Vieira et al. (131); Brazil δ, ε	95 MB leprosy (30LL/65BL) of which 51 ENL	At leprosy diagnosis and at onset of reactional episode	Time of MDT for each ENL	Sample before thalidomide and steroids?	PBMC	Lymphocyte transformation test (LTT)	Some patients showed lymphoproliferative response during ENL

Mahaisavariya et al. (103); Thailand	17 non-reactional, 8 T1R, 12 ENL	Biopsy at the time of diagnosis and not the time of reaction	ND	ND	Skin	T-lymphocyte subsets	%age of CD8 infiltration reduced in ENL compared with non-reactional lepromatous
							The CD4^+^/CD8^+^ ratio of ENL statistically significant higher than from the non-reactional lepromatous group

Tadesse et al. (132); Ethiopia δ	33 leprosy: 14 BT, 11 BT T1R, 8 ENL, 11 HC	ND	Certain leprosy patients were treated on MDT	All ENL treated with steroids	PBMC	Lymphocyte blast transformation	Thalidomide treatment did not alter the lymphoproliferative response to the mycobacterial antigens during ENL

Mohanty et al. (133); India	21 BL/LL ENL, 38 TT/BT/BL/LL, 29 BT/BL T1R, 19 HC	ND	ND	ND	PBMC	Immune responses against Stress proteins of *M. leprae* (lymphoproliferation)	ENL: no significant role of stress proteins except a heightened lymphoproliferative response to the 28 kDa antigen
					Serum		

Villahermosa et al. (134); Philippines δ, ε	22 ENL	Before thalidomide and at study weeks 3 and 7 during thalidomide	MDT continued during the study	Samples untreated for antireactional drugs and during thalidomide treatment	Blood	Lymphocyte proliferation assays (LPA) to phytohemagglutinin and concanavalin A	Low LPA values pre-thalidomide in both PBMC and whole blood
					PMBC		

Attia et al. (107); Egypt	38 leprosy: 6 ENL, 38 HC	Untreated samples	Untreated samples	Untreated samples; excluded patients on immunosuppressive drugs	Blood	Frequency of circulating Tregs; FoxP3 expression	Significantly lower frequency of Tregs but higher FoxP3 expression in ENL

Massone et al. (116); Brazil ε	20 leprosy: 3 ENL	Biopsies at the time of diagnosis	10, 12 and 13 months after beginning of MDT for LL	Untreated for antireactional treatment	Skin	Presence, frequency and distribution of Tregs	No statistical difference in FoxP3 expression between TT, BT, BL, and LL
							Significant increase in FoxP3 expression in T1R compared to ENL

Rada et al. (135); Venezuela ε	? ENL81 LL, 41 BL, 41 BB, 3% BT	ND	ND	ND	Blood	Cell-mediated immunological tests to mycobacterial proteins	T-lymphocyte proliferative response in reactional and non-reactional patients was negative

Saini et al. (136); India δ	21 MB: 16 ENL, 5 T1R	ENL blood during reaction and at 0.5 and 1 year after the onset of reaction	Duration of MDT described	ENL patients received steroids	PBMC	Lymphoproliferation of PBMC stimulated with *M. leprae*, recombinant Lsr2 and 6 synthetic peptides spanning the Lsr2	All patients with active ENL showed lymphoproliferation in response to peptides A and F

Abdallah et al. (109); Egypt δ	43 leprosy: 6 ENL, 40 HC	Untreated patients	Untreated samples	Untreated	Blood	Circulating Tregs	Tregs/Teffs lowest in ENL

Attia et al. (110); Egypt δ	43 leprosy: 6 ENL, 40 HC	Untreated patients	Untreated samples	Untreated	Blood	CD4(+) CD25(high)Foxp3 (+) regulatory cells	CD4(+)CD25(high)FoxP3(+) Treg levels lowest in ENL
							Treg/Teffs lowest in ENL

Hussain et al. (92); India	50 leprosy (28 without reactions, 11 T1R, 11 ENL), 50 HC, 50 pulmonary TB (25 HIV-TB co-infected and 25 without HIV infection), 50 HIV-positive	ND	Reactional episodes following antileprosy treatment	ND	Blood	CD3^+^, CD4^+^, CD8^+^ and CD4^+^/CD8^+^ ratio with flow cytometry	CD4^+^ counts raised during ENL compared to MB patients whereas CD8^+^ counts lower The CD4^+^/CD8^+^ ratio doubled during reactional episodes of T1R and ENL

Parente et al. (137); Brazil	2 ENL, 103 leprosy TT/BT/BB/BL/LL 9 indeterminate, 8 T1R	2 ENL: 12 and 10 months after initiation of MDT	2 ENL after initiation of MDT	ND	Skin	Frequency and distribution of regulatory T cells	No significant differences in ENL

Saini et al. (113); India δ	66 leprosy: 15 T1Rs, 15 ENL, 36 BT/LL	Newly diagnosed leprosy patients prior to institution of antireaction therapy	Freshly diagnosed patients: untreated subjects	Newly diagnosed leprosy patients prior to institution of antireaction therapy	PBMC	MLSA stimulated and unstimulated PBMC: gene expression with PCR array for 84 genes; T cell phenotypes	Increase in FOXP3 gene expression in ENL
							Th17 cells with intracellular IL-17A, F are increased in ENL and CD4^+^IL-21^+^ cells are higher in ENL
							Significant upregulation of CD4^+^CCR6^+^ cells in ENL
							Tregs decreased in ENL

*α, also in Table 1; β, also in Table 2; δ, also in Table 4; ε, also in Table 5*.

*BB, mid-borderline leprosy; BL, borderline lepromatous leprosy; BT, borderline tuberculoid leprosy; ENL, erythema nodosum leprosum; HC, healthy controls; HS, helper-suppressor; HSP, heat shock protein; ICs, immune complexes; LL, lepromatous leprosy polar; LTT, lymphocyte transformation test; LPA, lymphocyte proliferation assay; MB, multibacillary; ND, not described; PEG, polyethylene glycol; PHA, purified phhytohaemagglutinin; PPD, RT23 tuberculin-purified protein derivative; SLE, systemic lupus erythematosus; TB, tuberculosis; TT, tuberculoid leprosy polar*.

## What is the Role of TNF-α or Other Cytokines in ENL?

A role for TNF-α in ENL was first suggested by a Brazilian study that included 18 ENL patients at various stages of treatment with steroids or thalidomide (138). Serum TNF-α levels varied widely: from undetectable to extremely high levels (138). There was no obvious correlation between severity of ENL and cytokine levels, while patients who had received treatment had lower levels of TNF-α (138). High serum TNF-α levels were subsequently shown to decrease significantly during thalidomide treatment (139). These findings have been reproduced in other populations measuring serum TNF-α levels (128, 131, 140–147), whereas two studies failed to show increased levels of serum TNF-α during ENL (148, 149). The high variability in serum TNF-α between studies might be due to patient differences. Although genetic differences between different ethnic groups cannot be ruled out, it still remains unclear why there is such a high variability in the TNF-α levels between individuals presenting ENL.

A study of the plasma levels of TNF-α reported increased levels during ENL (150) while other studies contradicted this finding (115, 134, 151). In fact, Haslett et al., which included 20 male ENL patients excluding patients with moderate or severe ENL–associated neuritis, reported circulating plasma TNF-α levels to be lower at time of ENL diagnosis than LL controls (115). There was an upward trend in plasma TNF-α levels during thalidomide treatment which returned to baseline levels after discontinuation of thalidomide (115). This is an indication that thalidomide may in fact stimulate paradoxical overproduction of TNF-α (115). The inhibition of TNF-α by thalidomide may be prominent when macrophage production of this cytokine is high but in mild disease plasma levels may not reflect lesional TNF-α production (115). Increased TNF-α levels after thalidomide treatment has been described in other conditions such as toxic epidermal necrolysis (152) and aphthous ulcers in patients with human immunodeficiency virus infection (153). It has been suggested that the mechanism of the paradoxical overproduction of TNF-α by thalidomide could be due to the propensity of thalidomide to costimulate T-cells to produce cytokines including TNF-α (154). All the patients in the study of Haslett et al. showed improvement in ENL after receiving thalidomide during the first 21 days of treatment (115).

Interestingly, the studies that measured the *ex vivo* PBMC production of TNF-α in response to lipopolysaccharide, BCG, or *M. leprae* in patients with ENL as compared to BL/LL patients showed consistently greater amounts of TNF-α secretion in patients with ENL (150, 155–157).

The successful use of the anti-TNF therapy with infliximab and etanercept in three patients with ENL, resulting reduction of inflammation and treatment of ENL, is additional evidence of the inflammatory role of TNF-α in ENL (158–160).

The results of studies on IFN-γ are more consistent than those on TNF-α suggesting an important role for IFN-γ in the pathophysiology and occurrence of ENL. A clinical trial administered recombinant IFN-γ to BL/LL patients as a replacement therapy because LL is characterized by anergy to antigens of *M. leprae* and inability to produce IFN-γ (150). Repeated intradermal injection of recombinant IFN-γ induced ENL in 6 out of 10 BL/LL patients within 7 months compared to an incidence of 15% per year in patients who received MDT alone (150). Elevated serum IFN-γ was found in patients with ENL who also had high TNF-α levels (139). Other studies have demonstrated an increase of serum IFN-γ (143, 144, 148) and an increase of IFN-γ mRNA in PBMC (161–163) and in skin biopsies (161, 164) during ENL. There is a study reporting serum IFN-γ to be significantly lower in patients at the onset of ENL, which increased after thalidomide treatment (142). However, IFN-γ has been identified by Ingenuity Pathway Analysis networks as the second most significant upstream regulator (after CCL5) of the expression changes in microarrays performed in PBMC derived from patients with ENL (58).

There are contradictory findings about the role of serum IL-1β levels. Most studies have reported that serum IL-1β levels may have a prognostic value for developing ENL (144, 148, 165, 166) and that there is a statistically significant correlation between TNF-α and IL-1β (140). However, studies failed to show any association of serum IL-1β or plasma IL-1β with ENL (138, 151). IL-1β mRNA in PBMC was upregulated at the onset of ENL (161) but not in skin lesions (167).

IL-2 has a key role in the immune system primarily by its direct effects on T-cells such as promoting differentiation of different T-cell subsets and contributing to the development of T-cell immunological memory. IL-2 signals through the IL-2 receptor (IL2R), which is essential for the signaling in T-cells. There were no differences in the serum IL-2 or IL2 mRNA in skin biopsies between ENL and patients with LL (115, 148, 151). However, four studies reported an increase in soluble IL-2 receptor (sIL2R) levels (115, 131, 165, 168) or IL2Rp55 mRNA in PBMC (161) in patients with ENL.

Serum IL-6 (147, 151, 169, 170) and IL-6 mRNA in PBMC and skin (161) have been reported to be elevated during ENL. IL-6 tag single-nucleotide polymorphisms have been reported to be a risk factor for ENL (170) and IL-6 plasma levels were correlated with the IL-6 genotypes (170). A study reported increased serum IL-6 receptor (sIL6R) levels in ENL, which declined significantly after the completion of a corticosteroid treatment (143). However, other studies did not show associations of IL-6 serum levels with ENL (134, 139, 143).

An *ex vivo* study in PBMC isolated from ENL patients and LL controls showed a correlation of raised levels of cytokines IL-17A and its isomers as well as other Th17-associated cytokines IL-21, IL-22, and IL-23 with ENL (113). However, other studies failed to detect an association of ENL with serum IL-17 (110, 151, 171).

There are 49 studies measuring cytokines in ENL (Table 4), and the majority of these studies show a significant increase of the pro-inflammatory cytokines during ENL. TNF-α appears to be a regulator of the condition while there is substantial evidence supporting a role for IFN-γ as well. There is also evidence that other cytokines such as IL-1β and IL-6 or cytokine receptors such as sIL2R and sIL6R are also involved. Therefore, inhibitors of these molecules may be useful in a clinical setting. It is possible that genetic differences could account for differences observed between studies but methodological differences are also likely factors.

**Table 4:** **Human studies on ENL investigating cytokines**.

Reference; study site(s)	Study population	Timing of sampling	MDT status	ENL treatment status	Type of samples	Measures	Findings
Filley et al. (168); India ε	7 ENL	Before, during and after the episode	All patients on MDT	ENL treated with steroids and/or thalidomide	Serum	IL2R	IL2R increase during ENL

Rea and Modlin (102); USA γ	ND	ND	ND	ND	Skin	IL-2 positive and IFN-γ positive mRNA-bearing lymphocytes	IL2- positive lymphocytes prevalent in ENL and in tuberculoid lesions
							Cells expressing IFN-γ mRNA in ENL lesions slightly increased compared to lepromatous

Sarno et al. (138); Brazil	18 ENL, 39 BT/BL/BB/LL, 4 T1R	ND	16/18 patients on various stages of MDT/2 untreated	3 ENL on thalidomide and 7 ENL on prednisone; others untreated for reaction	Serum	Tumor necrosis factor (TNF)-α and IL-1	TNF varied from undetectable to extremely high levels in ENL
							No correlation between severity of ENL and cytokine level
							Neither TNF nor IL-1 correlate with number or duration of ENL episodes
							Treated patients with steroids or thalidomide lower TNF

Sehgal et al. (172); India	11 ENL, 14 T1R, 20 leprosy non-reactional, 10 HC	Before starting antireactional treatment and when clinical signs of reaction had abated	On MDT	Samples before and after starting antireactional treatment	Serum	IL-2R	T1R upgrading group higher IL-2R than ENL

Sullivan et al. (173); USA ε	ND	ND	ND	ND	Skin	IFN-γ and TNF-α mRNA	IFN-γ mRNA in ENL similar to tuberculoid
							In LL and ENL lesions about 0.2% of cells expressed TNF-α

Barnes et al. (155); USA	12 active ENL, 14 inactive ENL, 6 T1R; 11 LL	ND	All patients had received less than 5 years chemotherapy	ND	PBMC	TNF-α	ENL: the levels of TNF-α release by PBMC were higher than any other leprosy
							Thalidomide reduced TNF-α by more than 90%

Parida et al. (140); India	12 ENL, 64 leprosy TT/BT/BB/BL/LL, 14 T1R	ND	Most patients before MDT treatment	ND	Serum	TNF and IL-1	Patients undergoing T1R or ENL showed high TNF levels
							Significant correlation between TNF and IL-1 in reaction

Sampaio et al. (150); Brazil and USA	13 LL ENL, 15 LL, 9 HC	ND	All patients were receiving MDT during the study.	7 ENL patient blood samples before starting treatment with thalidomide and 6 1-2 weeks after thalidomide	Plasma	TNF-α	ENL patients greater release of TNF-α from monocytes
					PBMC		High plasma TNF-α in ENL
					Monocytes		

Bhattacharaya et al. (146); India	11 ENL, 14 T1R, 20 leprosy without reactions, 20 HC	Before treatment and after clinical remission of reaction	on MDT	Before antireactional treatment with steroids	Serum	TNF	TNF levels in acute ENL were higher but not significant and rose to become significant following treatment and clinical remission than HC and MB controls

Foss et al. (128); Brazil γ	28 lepromatous: 11 ENL, 23 tuberculoid, 19 HC	ND	86% of lepromatous patients treated with dapsone	Time of blood collection no immunosuppressive drug	Serum	TNF-α	TNF was elevated in the serum of ENL patients

Sampaio et al. (139); Brazil ε	49 BL/LL: 24 developed ENL	At the time of developing ENL, during thalidomide treatment, or after thalidomide treatment was discontinued; collected at 1-3, 6-7, and/or 13-21 days of thalidomide and 1-2 months after thalidomide	MDT was continued through the study	Thalidomide treatment for ENL	Sera	TNF-α, IL-6, IFN-γ	ENL highest TNF-α levels, which decreased significantly during thalidomide treatment Serum IFN-γ elevated in patients with high TNF-α levels

Santos et al. (156); Brazil	14 ENL (4 BL/10 LL), 12 BL/LL, 11 HC, 4 ENL post-reactions	ND	Half untreated and the other half treated with MDT	ENL patients were treated with thalidomide?	PBMC	TNF-α: spontaneous and *M. leprae* stimulated	ENL patients showed significantly greater release of TNF-α both spontaneously and induced by *M. leprae*-induced release in ENL patients

Vieira et al. (131); Brazil γ, ε	95 MB (30 LL/65 BL) of which 51 ENL	At leprosy diagnosis and at onset of reactional episode	Time of MDT for each ENL	Sample before thalidomide and steroids?	Serum	TNF-α, soluble IL-2R	TNF-α increased in 70.6% of ENL patients

Memon et al. (141); Pakistan	12 ENL, 27 leprosy (TT/BT/BL/LL), 14 household contacts and 22 endemic HC with no known leprosy contact	At the onset of ENL before initiation of treatment for reaction and after the reaction had subsided	10/12 ENL received previous MDT	Samples before antireactional treatment	Serum	TNF-α	TNF levels higher during acute phase of ENL and declined after clinical remission of the reaction

Moubasher et al. (148); Egypt	35 reactional (19 ENL/16 T1R), 55 leprosy, 20 HC	ND	Untreated ENL?	Untreated ENL?	Serum	IFN-γ, IL-2, IL-2R, IL-10, TNF-α, IL-1β	Both T1R and ENL showed significantly higher serum IFN-γ, IL-2R and IL-1β compared to non-reactional leprosy ENL showed increased levels of IL-10 compared to T1R

Moubasher et al. (165); Egypt	35 reactional (19 ENL), 36 non-reactional, 20 HC	PB patients assessed after 6 and 12 months of MDT/MB assessed after 12 months of MDT; Before and at the end of treatment with MDT	Before and after treatment with MDT	Corticosteroids were given to control the reactions	Serum	IL-2R, IL-10, IL-1β	IL-1β levels may have a prognostic marker for the development of reactions

Partida-Sanchez et al. (142); Mexico ε	9 ENL, 10 non-ENL, 10 HC	Beginning of reaction and after 1 and 2 months of thalidomide	All patients on MDT	Untreated samples and after 1 and 2 months of thalidomide	Serum	TNF-α, IFN-γ	TNF-α was significantly higher in ENL compared to non-ENL
							TNF levels decreased after ENL treatment
							IFN-γ significantly lower in patients at the onset of ENL and increased after thalidomide

Sampaio et al. (147); Brazil	18 MB with ENL (5BL/13LL)	Biopsies at diagnosis, at onset of reaction, and after 3 and/or 7 days of pentoxifylline; Serum: day 0 (during ENL), 3-7, 10-14, 30 and 60 days after pentoxifylline	7 patients with ENL newly diagnosed; others on MDT	Pentoxyfylline, 2 ENL patients on thalidomide	PBMC	Serum TNF-α, IL-6, IL-10	Elevated TNF-α in the sera of ENL
					Serum	TNF-α, IL-6, IL-10 release by PBMC following *M. leprae* stimulation or LPS stimulation	Treatment with pentoxifylline reduced TNF-α
							Serum levels of IL-6 increased during ENL
							High TNF-α mRNA expression in lesions during ENL which decreased following treatment with pentoxifylline
					Skin	TNF-α, IL-6, IL-10 gene expression at skin	IL-6 mRNA reduced by up to 50-fold after treatment

Moraes et al. (161); Brazil	53 leprosy: 20 ENL, 11 T1R	At the time of leprosy diagnosis (unreactional) and at the onset of first reactional episode (reactional)	MDT was continued through the study	No anti-inflammatory drugs at the time of sample collection	PBMC	IL-1β, IL-6, IL-8, GM-CSF, IFN-γ, IL-2Rp55, perforin, TNFβ, TNF-α mRNA in PBMC; IL-4, IL-6, IL-8, IL10, IL-12, IFNγ, TNFα mRNA in skin	In 7 ENL higher incidence of IFN-γ, perforin, GM-CSF, IL2R mRNA in blood
							Upregulation of IL-1β, IL-6, GM-CSF, IL-2R, IFN-γ mRNA in blood at onset of ENL at 3 ENL follow-up
				3 patients sequential sampling and after thalidomide	Skin		Skin lesions ENL: IFNγ and IL-4 differentially expressed

Oliveira et al. (33);Brazil α	10 BL/LL: 6 ENL, 10 HC	ND	On MDT	ND	Blood, P.B.Neutrophils	TNF-α, IL-8	Stimulated neutrophils secrete IL-8 and TNFα
							Increased TNF-α secretion from neutrophils after LPS stimulation
							Thalidomide inhibited TNF-α by neutrophils

Goulart et al. (174); Brazil	19 leprosy: 5 ENL/3 T1R, 9 HC	Untreated samples	Untreated samples	Untreated samples	PBMC	TGF-β1 in supernatants from adherent PBMC after stimulation with PGL-1, LPS or serum-free RPMI	Adherent PBMC from ENL secrete higher TGF-β1

Moraes et al. (164); Brazil	13 MB: 10 ENL, 3 T1R	Before and during pentoxyfilline or thalidomide	All patients on MDT	Before and during pentoxyfilline or thalidomide	Skin	mRNA expression: IFN-γ, IL-6, IL-10, IL-12 p40, TNF-α, IL-4	Expression of IFN-γ, IL-6, IL-10, IL-12 p40, TNF-α at the onset of reactional episodes (T1R and ENL) but IL-4 rarely detected Follow-up: TNF-α mRNA and IFN-γ, IL-6 and IL12p40 mRNA decreased after thalidomide or pentoxyfylline

Nath et al. (162); India	36 ENL, 105 TT/BL/LL 7T1R, 9 HC	ND	All patients on MDT	ENL patients before antireactional treatment	PMBC	IFN-γ, IL-4, IL-10, IL-12	ENL: 58% demonstrated a polarized Th1 pattern with only 30% expressing both cytokines

Nath et al. (163); India	1 BL/7 LL ENL, 2 BL/6 LL8 stable	ND	Most patients on MDT	ENL patients prior to antireactional therapy	PBMC	Real-time PCR forIFN-γ, IL4, IL10, p40 IL12	IFN-γ detectable in all and IL12p40 in half of ENL IL12p40 mRNA higher in ENL compared to stable lepromatous

Sampaio et al. (157); Brazil	15 leprosy: 10 ENL	ND	On MDT	ND	PBMC, monocytes, monocytes/T-lymphocytes cocultures	TNF-α after stimulation with *M. leprae*	Isolated monocytes from ENL released significantly more TNF-α in response to *M. leprae* than monocytes from non-reactional

Tadesse et al. (132); Ethiopia γ	14 BT, 11 BT T1R, 8 ENL, 11 HC	ND	ND	All ENL treated with steroids	PBMC	TNF-α in culture supernatants	Thalidomide resulted in suppression of TNF-α production

Haslett et al. (115); Nepal	20 ENL, 20 LL with no history of ENL within the preceding 30 days	Blood samples: days 0, 3, 7, 14, and 28 of thalidomide; ELISPOT: days 0, 7, 21, and 28	All (except 1 patient) on MDT	Excluded patients who had received immunomodulating therapy within the preceding month	Plasma	Plasma levels of IFN-γ, TNF-α, soluble IL2R, IL-12, IL-12 p40 and IL-12 p70	Circulating TNF-α levels lower at ENL diagnosis than controls
		Flow cytometry: days 0, 7, and 21; qRT-PCR: PBMCs days 0, 7, 21			T-cells	ELISPOT for IFN-γ; Flow cytometry for cytokine production by T cells	Upward trend during thalidomide ENL baseline plasma levels of IL-12 lower than control
					Skin	qPCR: IL-2 genes	Baseline levels of sIL2R higher in ENL than controls
							Thalidomide increased T cell subsets expressing both IL-2 and IFN-γ

Villahermosa et al. (134); Philippines γ, ε	22 ENL	Before thalidomide and at study weeks 3 and 7 during thalidomide	MDT was continued	Samples untreated for antireactional drugs and during thalidomide	Plasma	TNF-α, IL-6	TNF-α levels not detected
							IL-6 unchanged or reduced following thalidomide from week 0 to week 3
							IL-6 undetectable at weeks 3 and 7

Belgaumkar et al. (169); India	71 BT/BB/BL, 11 pure neuritic, 6 T1R, 1 ENL, 30 HC	Untreated samples	Untreated samples	Patients on antileprosy treatment or steroids were excluded	Serum	IL-6, IFN-γ	The one patient with ENL had higher levels of IL-6 and IFN-γ in comparison to the BL/LL patients without reactions

Iyer et al. (143); Indonesia ε	131 TT/BT/BB/BL/LL, 44 ENL, 5 T1R, 112 HC	ND	Patients on MDT	Prednisolone to treat reactions	Serum	IL-6, IFN-γ, TNF-α, IL-6R, IL-10, IL-4, sCD27	IFN-γ and IL-6R increased in ENL compared to non-ENL
							Completion of corticosteroid treatment: IFN-γ, TNF-α, sIL6R declined

Stefani et al. (151); Brazil	10 ENL, 10 T1R, 29 non-reactional controls	Newly detected untreated patients	Untreated samples	Untreated samples	Plasma	TNF-α, IFN-γ, IL12p70, IL-2, IL-17, IL-1β, IL-6, IL-15, IL-5, IL-8, MIP-α, MIP-β, RANTES, MCPI, CCL11/eotaxin, CXCL10, IL-4, IL-10, IL13, IL-1Rα, IL-7, IL-9, G-CSF, PDGF BB, bFGF, VEGF	IL-6, IL-7 and PDGF BB elevated in ENL

Motta et al. (175); Brazil	44 leprosy of which 15 ENL, 10 HC	Baseline and 7 days after therapy for oral infection	ND	ND	Serum	IL-1, TNF-α, IL-6, IFN-γ, IL-10	No specific finding for ENL

Teles et al. (176); Brazil ε	32 leprosy: 10 ENL, 8 T1R	4 ENL patients before and during reaction	All patients on MDT	ND	Skin	TNF-α gene expression and levels in supernatants	PBMC stimulated with *M. leprae*: upregulation of gene expression of TNF-α and increase of TNF-α in supernatants after 1, 3, and 6 h
					PBMC		

Jadhav et al. (149); India ε	303 MB: 5 ENL	Serum samples at the time of recruitment	Newly registered: no MDT	Untreated	Serum	TNF-α	No significant outcome for ENL

Madan et al. (144); India	61 leprosy: 4 ENL and 2 ENL during study	Untreated samples, during reactional episodes and after completion of treatment	Untreated patients	Patients on steroids were excluded	Serum	TNF-α, IFN-γ, IL-1β, IL-10	All cytokines were raised in reactional (both T1R and ENL) compared to non-reactional IFN-γ, IL-1β and IL-10 were higher in ENL but only IL-10 was statistically significant compared to T1R
							Levels of all cytokines decreased after MDT

Rodrigues et al. (145); Brazil	18 LL with ENL during treatment; 13 non-reactional BT, 37 non-reactional BL/LL, 25 BL with T1R during treatment; 21 HC	Beginning of leprosy treatment, at diagnosis of reactional episode and at 3-5 years post-treatment	Samples before and during MDT	Untreated samples and after treatment with prednisolone	Serum	TNF-α	TNF-α higher during ENL than prior to the reaction

Chaitanya et al. (177); India	21 ENL, 80 T1R, 80 leprosy without reaction, 94 non-leprosy	Untreated samples	Untreated samples	Untreated samples	Serum	IL-17F	IL-17F elevated during T1R but no significant difference in ENL

Lockwood et al. (178); India ε	303 MB leprosy: 13 ENL	Skin biopsies at enrollment	Before MDT	Before antireactional treatment	Skin	TNF-α and TGF-β immunostaining	TNF-α: similar levels ENL and non-ENL TGF-β: no difference in ENL and non-ENL

Martiniuk et al. (179); Nepal and USA ε	7 ENL	Pre- and post- treatment with thalidomide	ND	Pre- and post- treatment with thalidomide	Skin biopsies	RT-PCR for hIL-17A, hIL-17B, hIL-17C, hIL-17D, hIL-17E, hIL17F	IL17A, was consistently seen before and after thalidomide
							Reduction in IL17B, IL17E and increase of IL17C following thalidomide

Sousa et al. (170); Brazil	33 ENL, 54 T1R, 16 reaction-free leprosy	ND	63.8% presented ENL during MDT	ND	Plasma	IL-6	Higher IL-6 in ENL and T1R compared to non-reactional

Abdallah et al. (171); Egypt	43 leprosy: 6 ENL, 43 HC	Untreated samples	Untreated samples	Untreated samples	Serum	IL-17, IL-4	Overproduction of IL-4 in LL patients

Saini et al. (136); India γ	21 MB: 16 ENL, 5 T1R	ENL blood during reaction and at 0.5 and 1 year after the onset of reaction	Duration of MDT described	ENL patients received steroids	PBMC	PBMC stimulated with *M. leprae*, recombinant Lsr2 and 6 synthetic peptides spanning the Lsr2 sequence: IFN-γ	During ENL stimulated PBMC showed IFN-γ release

Abdallah et al. (109); Egypt γ	43 leprosy: 6 ENL, 40 HC	Untreated patients	Untreated samples	Untreated samples	Serum	IL-1β, IL-4, IL12p70, IFN-γ	IL-4 highest among LL compared to ENL

Attia et al. (110); Egypt γ	43 leprosy: 6 ENL, 40 HC	Untreated samples	Untreated samples	Untreated samples	Serum	IL-17, IL-22, IL-10, TGF-β	No statistically significant difference between groups

Berrington et al. (167); Nepal	85 leprosy: 9 ENL, 35 BL/LL non-reactional	ND	ND	ND	Skin	RT-PCR for CCL1, CCL2, CCL17, CCL18, IFNA1, IFNA8, IFNB1, IFNG, IL10, IL12a, IL12b, IL13, IL17a, IL18, IL1b, IL1ra, IL21, IL22, IL23, IL27, IL29, IL4, IL6, TNF	CCL18, IL12b and CD14 elevated in lesions of ENL but failed to reach significance when adjusted for multiple comparisons

Sallam et al. (166); Egypt	43 leprosy: 6 ENL, 43 HC	Untreated samples	Untreated samples	Untreated samples, excluded patients on corticosteroids	Serum	IL-1β, IL-12	Higher IL-1β in ENL compared to non- reactional
							No significant difference for IL-12

Dupnik et al. (58); Brazil β, ε	11 ENL, 11 T1R, 19 leprosy without reactions for microarray; 6 ENL, 11 T1R, 11 non-reactional for qPCR; 3 ENL for ICH	ND	3/11 ENL pre-treatment, 2/11 ENL on treatment and 6/11 post-treatment; leprosy controls matched for length of treatment	Excluded patients on steroids within 7 days and thalidomide within 28 days of enrollment	PBMC	Microarrays followed by qPCR	Cytokine-cytokine receptor interaction has been in the top 3 KEGG pathways in ENL CCL5 followed by IFN-γ was the most significant upstream regulator of the expression changes in the array

Saini et al. (113); India γ	66 leprosy: 15 T1R, 15 ENL, 36 stable leprosy without previous history or clinical evidence of reactions	Newly diagnosed leprosy patients prior to institution of antireaction therapy	Untreated samples	Untreated samples	PBMC	Antigen (MLSA) stimulated and unstimulated PBMC: gene expression with PCR array for 84 genes ELISA for cytokines IL-17A/F, IL-21, IL-22, IL-23A, IL-6, IL-1β, IFN-γ, TGF-β in supernatants	IL-23A mRNA expression increased in ENL IL-23R expression increased in ENL High expression of CCL20 and CCL22 in ENL ENL significant fold increase in IFN-γ Culture supernatants: Higher IL-17A/F in ENL patients compared to LL IL23A increased compared to LL IL-1β increased in ENL

Dias et al. (80); Brazil ε	30 ENL, 24 BL/LL, 31 HC	Upon diagnosis of reaction	BL/LL before MDT but most ENL patients on MDT	Before treatment with thalidomide or steroids	PBMC	TNF, IL-6 and IL-1β in response to TLR9 agonist	Higher production of TNF-α, IL-6, IL-1β in response to TLR9 agonist
							TLR9 antagonist inhibited the secretion of cytokines in response to *M. leprae* lysate

*α, also in Table 1; β, also in Table 2; γ, also in Table 3; ε, also in Table 5*.

*BB, mid-borderline leprosy; BL, borderline lepromatous leprosy; BT, borderline tuberculoid leprosy; ENL, erythema nodosum leprosum; HC, healthy controls; ICs, immune complexes; LL, lepromatous leprosy polar; ND, not described; P.B.neutrophils, peripheral blood neutrophils; SLE, systemic lupus erythematosus; TB, tuberculosis; TT, tuberculoid leprosy polar*.

## What Other Immune Mechanisms are Implicated in ENL?

Sixty-four studies on other immunological factors in ENL have been performed (Table 5).

**Table 5:** **Human studies on ENL investigating other immunological factors**.

Reference; study site(s)	Study population	Timing of sampling	MDT status	ENL treatment status	Type of samples	Measures	Findings
Waters et al. (22); United Kingdom and Malaysia β	38 lepromatous ENL	ND	ND	ND	Serum	Immunoglobulins	No differences in immunoglobulin levels

Reichlin et al. (180); Malaysia	13 LL of which 7 ENL	ND	ND	ND	Blood	Euglobulin IgG	Levels of euglobulin IgG higher in the ENL-positive patients than in ENL-negative patients
						Serum IgG	

Anthony et al. (60); India β	25 LL ENL, 10 LL without ENL	Active ENL lesions?	ND	ND	Serum	Immunoglobulins	High levels of immunoglobulins in both LL and ENL

Harikrishan et al. (71); India β	20 active LL; 15 ENL during active and subsided phase; 20 HC	ENL: during the active and subsided phase	ND	ND	Plasma	Immunoglobulins IgG, IgM, IgA	Serum levels of IgG and IgM during subsidence of ENL were significantly lower compared to that during the active phase of ENL

Humphres et al. (181); USA	14 LL ENL, 28 BL/LL, 21 HC	Multiple Serial sampling	10/19 LL patients untreated, 9/19 LL patients Dapsone	Corticosteroids day prior to initial assay for NK activity and was continued through treatment	PBMC	Natural killer cell activity	Natural killer cell activity significantly depressed in ENL

Rea and Yoshida (182); USA	108 leprosy (4 untreated ENL, 14 dapsone-treated active ENL, 10 dapsone-treated inactive ENL), 25 HC	ND	54 untreated patients and others dapsone-treated	Untreated	Blood	Macrophage migration inhibition activity	Positive serum inhibitory activity strongly associated with reactional states (ENL or T1R or Lucio’s reaction) in both treated and untreated patients

Miller et al. (183); USA	9 leprosy: 3 T1Rs and 2 ENL	Serial sampling from date of initiation of therapy until the first year of treatment	On MDT	Reactional episodes were treated with corticosteroids and 1 ENL received thalidomide	Plasma	Antibodies to Mycobacterial Arabinomannan	High levels of antibody to Arabinomannan in 2 ENL patients

Narayanan et al. (89); India γ	35 leprosy patients: 7 LL with ENL, 6 BT, 6 BT with T1R, 4 BL, 5BL with T1R, 8 LL	ND	ND	ND	Skin	B cells	No increase of B cells in any of the lesions

Rea et al. (96); USA γ	19 ENL, 67 BL/LL 4 LL with Lucio’s, 13 T1R, 18 Tuberculoid, 13 Tuberculoid long-term treatment	ND	Some patients on MDT	ENL before receiving thalidomide	PBMC	B-cells	B-cell percentage in the PBMC of ENL similar LL

Schwerer et al. (184); USA	121 leprosy (including ENL), 28 contacts, 15 HC	ND	ND	ND	Serum	Anti-PGL I IgM levels	Serum anti-PGL I IgM levels lower in ENL compared to patients with comparable BI

Andreoli et al. (40); India	12 ENL	ND	All patients on MDT with specified duration	Treated with prednisone and/or thalidomide	Serum	Circulatory IgM antibody levels to the PGL I; IgM, IgG, IgA antibody levels to *M. leprae* antigenic preparation	During ENL: decrease of circulatory IgM antibody levels to PGL I but no significant change to IgG, IgM or IgA antibody levels to the soluble antigens from *M. leprae*

Laal et al. (94); India γ	15 ENL, 13 LL	During active ENL and 1 week to 4 months after stopping treatment	Treatment with combination antileprosy drugs was continued throughout	First sample before initiation of antireactional treatment; second sample 1 week to 4 months after treatment	PBMC	B cells	B-cell percentages in PBMC of ENL patients were similar to those of uncomplicated LL

Blavy et al. (185); Senegal	34 ENL and 50 leprosy patients	ND	ND	ND	Lymphocytes	HLA phenotyping	Not significant findings of any HLA phenotype regarding ENL

Levis et al. (186); USA	ND	ND	ND	ND	Serum	IgM and IgG antibodies to PGL-I	ENL lower anti-PGL-I IgM than non-ENL of comparable BI

Rao and Rao (123); India γ	44 ENL, 39 LL, 22 post-ENL	ENL cases before starting treatment for ENL, post-ENL after the patient had not taken anti-inflammatory drugs or steroids for at least 3 and 7 days	From 39 non-reactional: 20 untreated and 19 with dapsone for less than a year	ENL before starting ENL treatment, post-ENL after the patient had not taken anti-inflammatory drugs or steroids for at least 3 and 7 days	Blood	B lymphocytes in peripheral blood	B cells: no difference between groups

Sehgal et al. (76); India β	21 patients with leprosy reactions either T1R or ENL	ND	ND	ND	B-cells	Percentage and absolute count of B-cells; Immunoglobulins IgG, IgA, IgM	During ENL a significant increase in the percentage and absolute count of B-lymphocytes Significantly elevated serum immunoglobulin values after subsidence of ENL
					Serum		

Levis et al. (187); USA	40 ENL, 63 leprosy without ENL, HC	ND	ND	ND	Serum	IgM antibody to PGL-I; IgM and IgG Abs to *M.tb* and *M. leprae* LAM	No correlation between IgM or IgG Ab to LAM and bacillary index

Rao and Rao (85); India	44 ENL, 39 lepromatous, 22 post-ENL	ENL blood before starting ENL treatment, post-ENL after patient does not take any anti-inflammatory drugs or steroids for the last 3 or 7 days	20 patients no previous MDT and 19 treated with dapsone	Before starting treatment with anti-inflammatory drugs or steroids	Blood	Leukocyte migration inhibition test	Lower migratory indices to whole *M. leprae* during ENL

Rao and Rao (78); India β	44 ENL, 39 BL/LL, 22 post-ENL	ENL before starting anti-ENL treatment, post-ENL ensuring that the patient had not taken anti-inflammatory drugs or steroids for at least 3 or 7 days	20 BL/LL untreated and 19 BL/LL treated with dapsone	Before starting treatment with steroids or anti-inflammatory drugs	Serum	IgG, IgA, IgM	IgG and IgM decreased in ENL than lepromatous and post-ENL
							Serum IgA elevated in ENL than lepromatous group and further increase post-ENL

Filley et al. (168); India δ	7 ENL	Before, during and after the episode	All patients on MDT	ENL was treated with steroids and/or thalidomide	Serum	%GO	During ENL%GO transiently raised, and this rise parallels an increase in circulating IL2R

Bhoopat et al. (127); Thailand γ	57 ENL (19 acute/38 chronic), 61 active LL, 33 control patients whose leprosy had been cured	26 BL and 35 LL newly diagnosed and untreated	ND	When treatment with corticosteroids and/or thalidomide was initiated precise timing was recorded with respect to the time of collection of specimens	Blisters induced over a representative skin lesion	IgM antibody to PGL-I and Tac peptide	IgM antibody to PGL-I and Tac peptide levels were elevated in chronic ENL lesions
							Corticosteroids reduced IgM antibody to PGL-I but did not change the levels of Tac peptide

Ramanathan et al. (49); India β	26 BL/LL of which 11 ENL, 24 HC	Blood was taken before initiation of treatment and then to 2-month intervals up to 20 months	Untreated and then on MDT samples every 2 months	Treated but after blood sampling	Serum	IgG, IgA and IgM	ENL no significant relation with immunoglobulin levels

Sullivan et al. (173); USA δ	ND	ND	ND	ND	Skin	ICAM-1, ICAM-1 ligand LFA-1	Prominent keratinocyte ICAM-1 expression

Scollard et al. (82); Thailand β	4 cured leprosy, 10 leprosy (5BT, 3BL, 2LL), 8 ENL patients (5LL and 3BL), 3 T1R, 4 HC	ND	ND	ND	Blisters induced over representative skin lesion	Immunoglobulins (IgG, IgA, IgM) to whole *M. leprae and to* PGL-I	No statistically significant difference regarding immunoglobulins
					Sera		

Sehgal et al. (188); India	25 leprosy with reactions (of which 11 ENL), 20 leprosy without reactions, 10 HC	ND	On MDT	Reactional patients on prednisolone	Lymphocytes	Lymphocyte adenosine deaminase activity (L-ADA)	The patients with leprosy reactions (both ENL and T1Rs) had higher enzyme l-ADA than controls (the enzyme has a role in activation, differentiation and proliferation of lymphocytes)

Sampaio et al. (139); Brazil δ	49 BL/LL of which 24 ENL	ENL at the time of developing ENL, during thalidomide treatment, or after thalidomide treatment	MDT was continued through the study	Certain ENL during thalidomide treatment	Skin biopsies	MCH II and ICAM-1 in histology	MHC II and ICAM-1 on epidermal keratinocytes in ENL downregulated with thalidomide

Santos et al. (129); Brazil γ	10 ENL, 59 LL/BL, 4 T1R, 4 post-reactional	ND	On MDT	No antireactional treatment before blood collection	PBMC, Monocytes	Monocyte activation by procoagulant activity, HLA-DR	No significant difference in monocyte activation between the different groups No significant differences in HLA-DR between groups

Singh et al. (189); India	44 active ENL, 48 prior history of ENL, 125 stable lepromatous, 40 HC not endemic	ND	ND	Untreated samples	Serum	Antibodies against B cell epitopes of *M. leprae* recombinant protein LSR	Antibodies against a specific distinct peptide region only in patients undergoing ENL

Kifayet and Hussain (190); Pakistan	67 BL/LL acute ENL, 83 non-reactional BL/LL, 77 endemic HC	ND	Most on MDT but 83 non-reactional less than 2 weeks of MDT	ND	Plasma	*M. leprae*-specific IgG subclasses	Lower concentrations of all IgG subclasses during ENL but lower IgG1 and IgG3 during ENL before treatment

Kifayet et al. (191); Pakistan	13 ENL acute and post-remission of reaction, 16 non-reactional stable LL, 32 endemic HC	During acute ENL (*n* = 13) and after the reaction has subsided	ND	ND	Plasma	IgG subclasses *M. leprae*-specific antibodies; Detection and enumeration of antibody-secreting B cells by ELISPOT	Polyclonal IgG1 elevated in acute ENL compared LL controls and decreased when ENL subsided IgG2 antibodies lower during acute ENL and increased after reaction has subsided Discrepancy in serum concentrations and B cell frequency
					B-cells		

Vieira et al. (131); Brazil γ, δ	95 MB leprosy (30 LL and 65 BL) of which 51 ENL	At leprosy diagnosis and at onset of reactional episode	Time of MDT for each ENL patients in study	Sample before thalidomide and steroids?	Serum	Circulating anti-neural and antimycobacterial antibodies	Detection of anti-neural (anti-ceramide and anti-galactocerebroside) antibodies in ENL sera
							No difference between reactional and non-reactional lepromatous patients regarding IgM antibodies
							Higher levels of anti-ceramide IgM and diminished levels of anti-galactocerebroside antibodies in reactional compared to non-reactional patients

Rojas et al. (50); Brazil β	19 ENL, 10 BL/LL non-ENL patients, 13 family contacts; 15 healthy non-contacts	ND	Both untreated patients and patients on MDT for 1-72 months	ND	Serum	Anti-PGL-I IgM, IgG responses to recombinant 10-kDa heat shock protein	IgM anti-PGL-I and IgG anti-10-kDa heat shock protein antibodies were constituents of the immune complexes in patients with ENL while free antibody levels did not differentiate between ENL and non-ENL patients

Beuria et al. (192); India	18 ENL, 44 BL/LL, 62 BT/TT, 17 HC	ND	Most patients on MDT	ND	Serum	IgG subclass levels to *M. leprae* sonicated antigens (MLSA) and PGL-I	ENL patients showed a significant fall in IgG3 antibody to MLSA and PGL-I compared to BL/LL leprosy controls

Freire et al. (193); Brazil	59 leprosy (including 12 ENL), 60 HC	ND	11/12 ENL were on MDT and 1/12 with dapsone	ND	Serum	Anti-neutrophil cytoplasmic antibodies (ANCA)	ANCA are present in 28.8% of leprosy patients but are not related to vasculitis in the ENL reaction and are not a marker of a specific clinical from

Partida-Sanchez et al. (142); Mexico δ	9 ENL, 10 non-ENL leprosy, 10 HC	Beginning of reaction and after 1 and 2 months of thalidomide	All patients on MDT	Before thalidomide, second sample after 1 month of thalidomide and third after 2 months	Plasma	IgM and IgG antibody subclasses to *M. leprae* sonicated extract	ENL at the onset of reaction had slightly higher anti-*M. leprae* IgG1 and IgG2 antibodies compared to non-ENL but not statistically significant

Stefani et al. (194); Brazil	600 leprosy: 31 ENL, 45 T1R, HC	Untreated	Before MDT treatment	Untreated	Serum	IgM and IgG anti-PGL-I	Patients presenting with T1R or ENL at leprosy diagnosis have same level of IgM anti-PGL-I antibody response as leprosy patients without reactions at diagnosis

Beuria et al. (195); India	44 BL/LL, 62 TT/BT, 18 ENL, 15 T1R, 17 HC	ND	BL/LL: 90% on MDT	Steroids after collection of samples	Serum	IgG1, IgG2, IgG3 and IgG4 to LAM	Reduction in IgG3 in ENL compared to active BL/LL Higher IgG1 in ENL than T1R
			TT/BT: mostly untreated				

Hamerlinck et al. (196); Philippines, Netherlands	13 ENL, 22 T1R, 26 leprosy unreactional, 10 HC	Serial samples during MDT: 2 ENL follow-up and received corticosteroids, 14 leprosy free of reactions, 4 T1R 6, 12, 18, 30 months during follow-up	13 ENL before MDT, 2 ENL during MDT	During ENL before treatment	Serum	Neopterin	T1R and ENL higher neopterin levels compared to non-reactional individuals Corticosteroid treatment reduces levels of neopterin

Mahaisavariya et al. (197); Thailand	95 leprosy patients: 63 non-reactional, 19 T1R, 13 ENL	A biopsy at time of diagnosis and an additional biopsy later, in some cases at the time of reaction	ND	Before antireactional treatment?	Skin	Mast cells	Reduction of mast cell counts in both T1R and ENL compared to non-reactional patients

Schon et al. (198); Ethiopia	4 ENL, 5 T1R	ND	ENL cases: 2 MDT untreated and 2 MDT-treated	Steroids	Urine	Urinary levels of metabolites of NO	Urinary nitric oxide metabolites decreased significantly after steroid treatment

Antunes et al. (199); Brazil	3 ENL, 3 T1R	First biopsy during reactional episode and second during remission	On MDT	Thalidomide for ENL	Skin	Neuropeptides; quantification of mast cells and their subsets	Increase mast cells in the inflammatory infiltrate of the reactional (both T1R and ENL) biopsies compared to non-reactional

Rada et al. (200); Venezuela	29 ENL, 19 MB not reactional, 11 PB, 28 HC	Before treatment	Untreated samples	Untreated samples	Serum	Nitrite/Nitrate levels	Supernatants of PBMC from ENL patients significantly elevated levels of nitrite/nitrates compared to LL or tuberculoid leprosy
					PBMC		

Sunderkotter et al. (201); Brazil	Skin Biopsies: 41 non-reactional leprosy, 8 ENL, 10 T1R; Serum samples: 16ENL, 5RR, 7TT, 13BT/BB/LL, 19 HC	Untreated samples	Skin biopsies: 42 untreated non-reactional leprosy	Before treatment with steroids or thalidomide	Serum Skin	MRP8, MRP14	Increase of serum levels of MRP8 and MRP14 in ENL Higher percentage of MRP8^+^ and MRP14^+^ cells in ENL skin lesions than non-reactional
			8 ENL of which 5 MDT				

Nigam et al. (202); India	80 leprosy: 10 ENL and 10 T1R; 20 HC	ND	ND	ND	Serum	Deaminase	Deaminase levels were higher in patients with reaction

Villahermosa et al. (134); Philippines γ, δ	22 ENL	Before thalidomide administration and at study weeks 3 and 7 during thalidomide treatment	MDT was continued during the study	Samples untreated for antireactional drugs and during thalidomide treatment	Urine	Neopterin	ENL higher neopterin values in urine than HC

Iyer et al. (143); Indonesia δ	131 leprosy patients (44 ENL), 112 HC	ND	Patients were classified irrespective of MDT status	Prednisolone to treat reactions	Plasma	Neopterin	Neopterin no significant difference between ENL and non-ENL

Mohanty et al. (203); India	14 ENL before and after resolution of ENL, 5 LL	Before commencing antireactional therapy and after resolution of ENL	All patients on MDT	Before commencing antireactional treatment	Urine	Urinary nitric oxide metabolites	Urinary nitric oxide metabolite higher in ENL compared to non-reactional LL
							These levels were reduced with resolution of reaction following antireactional therapy

Santos et al. (204); Brazil	8 leprosy: 3 ENL	ND	MDT during the study: length of MDT described	Thalidomide during the study	PBMC	B7-1 expression (flow cytometry and IHC)	Higher B7 expression in ENL and T1R patients than non-reactional in both PBMC and cutaneous lesions
					Skin		

Silva et al. (205); Brazil	25 leprosy: 5 ENL and 8 T1R	0, 2, 4, 6, and 12 months of MDT	All patients on MDT	Untreated	Plasma	PGL-I levels	Serum PGL-I levels did not differ significantly between ENL and non-ENL
						Neopterin	No significant correlation of neopterin between ENL and non-ENL

Brito Mde et al. (206); Brazil	104 reactions after completion of MDT (44 ENL), 104 with no post-treatment reactions (8 ENL)	ND	All patients were treated with MDT; half had finished MDT	ND	Plasma	ML flow (IgM anti-PGL-I positive serology)	The patients with positive serology after MDT presented a 10.4 fold greater chance of developing post-treatment reactions (ENL or T1R)

Iyer et al. (207); Indonesia	78 leprosy (36 ENL and 3 T1R), 36 HC	ND	30 untreated and 48 treated patients	Reactions were treated using prednisolone	Serum	Chitotriocidase	Serum chitotriosidase activity elevated in ENL compared with HC but not with non-ENL leprosy
					Skin		Significant decline of serum chitotriosidase following corticosteroid treatment in ENL

Lee et al. (24); USA α	6 ENL, 7 LL	ND	ND	ND	Skin	Microarrays and gene expression; IHC for E-selectin	Upregulation of gene expression: in ENL lesions of the selectin family of adhesion molecules
							IHC: higher levels of E-selectin in ENL lesions

Massone et al. (116); Brazil γ	20 leprosy biopsies (3 ENL)	Biopsies at the time of diagnosis	10, 12 and 13 months after beginning of MDT for LL	Untreated	Skin	Presence, frequency and distribution of plasmacytoid dendritic cells	CD123 expression was observed in 2/3 ENL biopsies

Rada et al. (135); Venezuela γ	81 LL, 41 BL, 41 BB, 3% BT	ND	ND	ND	Blood	Serological immunological tests to various mycobacterial proteins	Mean antibody values against complete mycobacterial proteins higher in non-reactional individuals

Teles et al. (176); Brazil δ	32 leprosy: 10 ENL, 8 T1R	4 ENL patients before and during reaction biopsy samples	All patients were receiving MDT	ND	Skin	MMP-2, MMP-9, TIMP-1	RT-PCR for MMP-2 and MMP-9 versus TIMP-1 in ENL sequential samples in 4 ENL patients: TNF-α, MMP-2 and MMP-9 mRNA enhanced IHC and confocal microscopy: absence of MMP positivity in ENL epidermis ELISA in sera of ENL: elevated MMP-9 but not TIMP-1 compared to non-reactional patients
					Serum		

Jadhav et al. (149); India δ	303 MB followed up for 2 years: 5 ENL	Serum samples at the time of recruitment	Newly registered MB patients: no MDT	Untreated	Serum	Antibodies to PGL-I, LAM, ceramide, S100	No statistically significant outcome for ENL

Lockwood et al. (178); India δ	303 new MB leprosy (13 ENL)	Skin biopsies at enrollment	Before MDT treatment started	Before antireactional treatment	Skin	Immunostaining for CD68 and iNOS	Reactional biopsies had significantly fewer CD68^+^ cells than non-reactional
							Nearly all biopsies in the LL group had CD68^+^ cells present and these were not altered in ENL
					Nerve		ENL showed some iNOS staining but not significant differences with non-ENL

Martiniuk et al. (179); Nepal and USA δ	7 ENL	Pre- and post-treatment with thalidomide	ND	Pre- and post- treatment with thalidomide	Skin	RT-PCR for hRORγT, hCD70, hCD27, hPLZF-1, hCTLA4, hAHR, hiNOS2, hARNT, hIDO, hGARP, hCD46	Reduction in CD70, GARP, IDO and increase of RORγT, ARNT following thalidomide treatment

Singh et al. (208); India	240 leprosy: 19 ENL, 69 BL/LL	ND	ND	ND	Serum	IgG antibodies against keratin	No significant difference in ENL

Dupnik et al. (58); Brazil β, δ	11 ENL, 11 T1R, 19 leprosy controls without reactions for microarray; additional 28 leprosy (6 ENL, 11 T1R, 11 non-reactional) for qPCR validation; 3 ENL for IHC	ND	3/11 ENL pre-treatment, 2/11 ENL on treatment and 6/11 post-treatment; leprosy controls matched for stage of treatment	Excluded patients on steroids within 7 days and thalidomide within 28 days of enrollment	PBMC	Microarray and qPCR for transcriptional profile of PBMC; Flow cytometry for monocyte populations	Top 3 KEGG pathways in ENL were *S.aureus* infection, SLE, cytokine-cytokine receptor interaction
							No significant difference in the proportion of circulating monocytes between reactional and non-reactional PBMC

Mandal et al. (209); India	15 reactional (both ENL and T1R), 15 HC	ND	ND	ND	PBMC	Vitamin D receptor (VDR) mRNA	All the individuals with low VDR expression manifested ENL

Dias et al. (80); Brazil δ	30 ENL, 24 BL/LL, 31 HC	Upon diagnosis of reaction	BL/LL before MDT but most ENL on MDT	Before treatment with thalidomide or steroids	PBMC (monocytes, B-cells, pDCs)	Expression of TLR9	Skin lesions and PBMC of ENL express higher levels of TLR-9
					Skin		

Schmitz et al. (25); Brazil α	62 leprosy: 22 ENL, 16 HC	ENL: before and 7 days after thalidomide	Patients before and after MDT	Before and after thalidomide	Skin	CD64 expression by qPCR and IHC	CD64 mRNA and protein expressed in ENL lesions
							Thalidomide reduced CD64 expression

*α, also in Table 1; β, also in Table 2; γ, also in Table 3; δ, also in Table 4*.

*BB, mid-borderline leprosy; BL, borderline lepromatous leprosy; BT, borderline tuberculoid leprosy; ENL, erythema nodosum leprosum; HC, healthy controls; ICAM-1, keratinocyte intracellular adhesion molecule 1; ICs, immune complexes; LAM, lipoarabinomannan; LL, lepromatous leprosy polar; MLSA, M. leprae sonicated antigens; ND, not described; PGL I, phenolic glycolipid I; SLE, systemic lupus erythematosus; TB, tuberculosis; TT, tuberculoid leprosy; polar,%GO, proportion of oligosaccharide chains on the Fc fragment of IgG which terminate with N-acetylglucosamine and not galactose*.

### Innate Immunity

Genetic studies have shown associations between several single-nucleotide polymorphisms (SNP) of innate immunity genes such as *NOD2* (210), the natural resistance-associated macrophage protein (*NRAMP1*) (211), and *TLR1* (212, 213) with ENL.

A recent study from Brazil, which investigated whether DNA sensing *via* TLR9, constitutes a major inflammatory pathway during ENL (80) showed that both the skin lesions and peripheral leukocytes (B-cells, monocytes, and plasmacytoid dendritic cells) of ENL patients express higher TLR9 levels than BL/LL controls (80). In addition, the levels of endogenous human and pathogen-derived TLR9 ligands (human and mycobacterial DNA-histone complexes) were also higher in the circulation of ENL patients than BL/LL controls (80). Furthermore, stimulation of PBMC isolated from ENL patients with TLR9 agonist led to higher levels of TNF-α, IL-6, and IL-1β, than those of non-reactional leprosy and healthy controls. Usage of a TLR9 synthetic antagonist was able to significantly inhibit the secretion of pro-inflammatory cytokines after stimulation with *M. leprae* lysate (80). This is the first study to support the potential of TLR signaling inhibitors as a therapeutic strategy for ENL (80).

### B-Lymphocytes and Immunoglobulins

Early studies enumerated B lymphocytes in skin lesions (89) and in peripheral blood (76, 78, 94, 123) of patients with ENL, while most of these studies did not find any association between B-cells and the development of ENL. Other studies looked at the IgM PGL-I in sera as a marker for ENL (40, 184, 192, 206), but most of these studies did not show an association (50, 82, 149, 187, 194, 205). Significantly lower serum levels of IgG1 and IgG3 subclasses of *M. leprae-*specific antibodies have been demonstrated in ENL patients compared to the BL/LL controls (190). This decrease of *M. leprae*-specific IgG1 and IgG3 antibodies in sera has not been related to downregulation of B cell responses since ENL episodes were characterized by an increase of polyclonal IgG1 antibody synthesis by the B cells, declining after subsidence of the reaction (191). The authors suggested that activation of B-cells is restricted to IgG1-secreting B cells in the blood of patients with lepromatous disease (191), while the lower serum concentrations of *M. leprae*-specific IgG1 and IgG3 (190) could be due to antibody deposition in the tissues (191). Interestingly, surface CD64 (FcγRI), the high-affinity receptor for monomeric IgG1 and IgG3 is expressed at higher levels on circulating neutrophils derived from ENL patients compared to non-reactional leprosy controls (25). The higher CD64 neutrophil expression could explain the presence of lower serum IgG1 and IgG3 levels in ENL patients compared to BL/LL controls.

### New Suggested Pathogenetic Mechanisms

Two recent studies of gene expression provide evidence of activation of novel molecular pathways in ENL.

Lee et al performed bioinformatic pathways analysis of gene expression profiles in leprosy skin lesions and found “cell movement” as the top biological pathway characterizing ENL (24). The study further described a neutrophil recruitment pathway including genes of key molecules that mediate neutrophil binding to endothelial cells (24). This neutrophil recruitment pathway characterizing ENL was inhibited by thalidomide (24). Consistent with these findings is a study of transcriptional profiles in PBMC of leprosy patients by Dupnik et al which identified “granulocyte adhesion and diapedesis” as one of the top canonical pathways characterizing ENL (58). Dupnik et al. identified 517 differentially expressed genes in patients with ENL (58). The pathway analysis revealed that the top three Kyoto Encyclopedia of Genes and Genomes (KEGG) pathways that changed in ENL were *Staphylococcus aureus* infection, systemic lupus erythematosus (SLE), and cytokine-cytokine receptor interaction, while the complement and coagulation pathway was also associated with ENL (58). CCL5 was the most significant upstream regulator in the array followed by IFN-γ (58). Transcripts uniquely increased in ENL included the complement receptors C3AR1 and C5AR1 while uniquely decreased transcripts in ENL included IL-10 and cytotoxic T-lymphocyte associates protein 4 (CTLA-4), modulators of T-cell responses (58). Hepcidin, catholicidin, antimicrobial peptides, C1q, and defencins had also an increased expression in ENL, while CCL2, CCL3, and SOD2 could be potential biomarkers for ENL (58). Transcripts increased in PBMC from ENL patients also included FcγR1 (CD64), FPR1, and FPR2, which recognize formylated peptides produced by bacteria triggering receptor on myeloid cells 1 (TREM1) and the related molecule triggering receptor expressed on myeloid cells-like 1 (TREML-1) (58).

The microarray studies performed in skin lesions and PBMC have generated a long list of candidate genes that regulate immune function to be associated with ENL. These merit further research.

## Limitations of the Systematic Review

PubMed was the only database used to identify eligible studies. This will have resulted in studies published in journals not listed in PubMed being omitted from our review. A search of gray literature may also have contributed data which may have influenced our conclusions. The high heterogeneity of included studies in terms of study questions and outcomes and the different methodologies used meant that a meta-analysis was not possible.

## Methodological Considerations of the Studies Included in the Systematic Review

Many of the studies of immunological features of ENL contain significant limitations in both design and reporting. Most seriously 66% of the studies did not have a case definition of ENL.

More than 70% of studies sampled individuals at a single time point. Sampling at two time points was seen in 21.2% of studies, 3 time points in 2.7% of studies, whereas 4 or more time points was described in only 5.5% of studies. Some studies did not have appropriate controls- patients with uncomplicated BL and LL. Although 93.2% of studies used BL/LL patients as controls, the remaining 6.8% of studies used other control groups such as healthy volunteers or leprosy contacts or tuberculoid leprosy patients or patients with Type 1 reaction. Often the controls were not matched for age, sex or treatment status. Controls should be matched for age and sex since these factors may influence T cell and neutrophil numbers and functions (214–216) as well as TNF-α and other cytokine levels (217).

ENL is a condition that can be acute, recurrent, or chronic, and therefore, the timing of sample collection is crucial. No information on the timing of the sampling is described in 54.8% of all studies. The importance of timing for sample collection during ENL could explain the discrepancies observed in multiple studies as has been suggested in the studies addressing the role of neutrophils in ENL. Studies using serial sampling yield more meaningful data compared to cross-sectional studies. The interval between time points is important and needs to be kept as consistent as possible for all study subjects.

Only one study matched BL/LL controls and ENL cases for length of MDT. Patients may develop ENL prior to the diagnosis of leprosy, during MDT or after successful completion of MDT. MDT may affect the immune status of leprosy patients and thus the matching of cases and controls for this variable is important. Two of the components of MDT, dapsone and clofazimine, have been associated with alterations in neutrophil and lymhocyte function (218–220). Dapsone stimulates neutrophil migration (218) and inhibits production of Prostaglandin E_2_ by neutrophils (220). In addition, dapsone inhibits lymphocyte transformation (218). On the other hand, clofazimine enhances production of Prostaglandin E_2_ by neutrophils (220). Dapsone and anti-dapsone antibodies have been identified in circulating ICs of leprosy patients (221). Circulating cytokine and chemokine levels also change with MDT (165, 222, 223). In addition, gene expression studies could be affected by MDT since the MDT component rifampicin may modify the expression of certain housekeeping genes (224). A total of 30.8% of studies did not report the MDT status of their cases or controls, 12.3% collected untreated patient samples, whereas 56.2% collected patient samples at various stages of MDT.

The effect of immunosuppressive drugs used to treat ENL on the findings of studies is an important factor which should be considered. In 37.7% of studies, there was no reporting of whether participants were on ENL treatment when samples were collected. Treatment with corticosteroids affects T-cells and neutrophil function (225, 226) and also gene expression studies by influencing housekeeping genes (224). Treatment with thalidomide may increase the neutrophil numbers, at least partially through differentially modulating the surface expression of markers CD18 and CD44 by the neutrophils in the bone marrow and the spleen (227). Thalidomide treatment may also affect T-cell functions by suppressing CD4^+^ T-cell proliferation while increasing their conversion to CD4^+^FoxP3^+^ Tregs (228). Moreover, thalidomide treatment may reduce cytokine levels (229). Less than half (34.2%) of studies indicate that samples were obtained prior to the start of ENL treatment.

Only 17.8% of all studies collected samples from more than one system, while samples from both blood and skin were described in only 12.3% of all studies.

## Future Study Design

Studies of ENL may be difficult to design and conduct. In addition, no animal model of ENL is available. Obtaining sufficient numbers of patients so that studies are adequately powered is difficult unless multicenter studies are performed which increase the logistical complexity and cost of the research. Patients are often on treatment (both MDT and immunosuppression) which may influence the study outcomes.

A large cohort study of newly diagnosed patients with BL and LL would be optimal in allowing matching of cases and controls. Some BL/LL patients who have not developed ENL at enrollment in the study should be recruited and followed until they develop the disorder. Detailed clinical information which includes demographic data, ENL severity using a robust measure, treatment status, in conjunction with well-timed and documented specimen collection (preferably of blood and skin), effective specimen storage, and transportation. ENL is a systemic disease and ideally samples from more than one system, i.e., both blood and skin should be obtained where appropriate. Well-designed laboratory experiments using a wide range of techniques should be used to interrogate such important specimens.

## Conclusion

**Figure 2:**
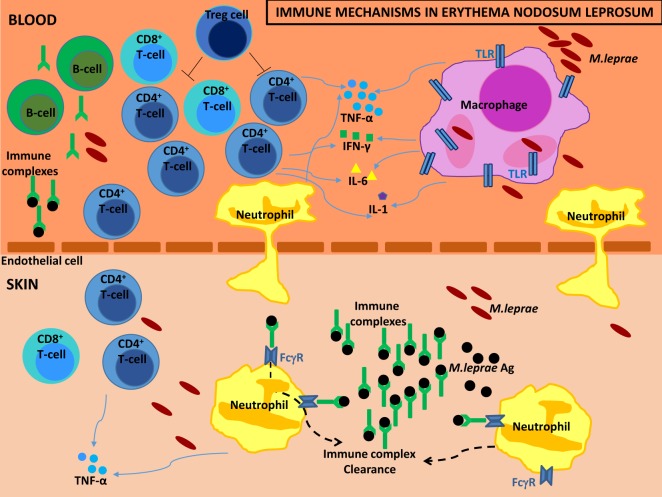
**Immune mechanisms in erythema nodosum leprosum (ENL)**. The diagram illustrates the different immune mechanisms which have been described in the literature of ENL. High volume of immune complexes (ICs) are formulated due to the increased antibody formation by the B cells and the increased mycobacterial antigens by fragmentation of the *M. leprae* bacilli. ICs are deposited in the skin. Neutrophils are drawn to the skin where they help in the IC clearance using their surface Fcγ receptors. An increase of CD4^+^/CD8^+^ T cell subset ratio in both peripheral blood and skin characterizes the disorder. Macrophages form the *M. leprae* intracellular niche and in concert with neutrophils and T-cells secret high levels of tumor necrosis factor (TNF)-α and other pro-inflammatory cytokines to further complicate the phenotype of ENL.

Figure 2 gives an overview of the immunology of ENL.

Our understanding of the causes of ENL is limited. The factors that initiate and/or sustain it might help to identify strategies to prevent or control the associated inflammation.

There is some evidence to support a role for neutrophils and ICs/complement in the inflammation associated with ENL; however, their role in the initiation of ENL remains unclear. The increase of TNF-α and other pro-inflammatory cytokines during ENL has been shown in multiple investigations, while suppression of TNF-α leads to clinical improvement. T-cell subsets appear to be important in ENL since multiple reports describe an increased CD4^+^/CD8^+^ ratio in ENL patients compared to BL/LL controls.

New technologies such as microarray studies pave the way and may lead to novel immunological pathways associated with ENL. Further research of the association of ENL with pathophysiological pathways such as the SLE pathway or the *S. aureus* infection pathway may improve our understanding of the disorder and potentially lead to novel therapeutic strategies. There are still large gaps in our understanding of this severe complication of leprosy despite the large number of studies examining the immunology of ENL. A systems biology approach may provide new insights.

This systematic review has highlighted the complex interactions at play in ENL and the difficulty in elucidating the various inflammatory pathways. We should rise to the challenge of understanding how these mechanisms operate and interact so that we can improve the treatment of patients with ENL.

## Author Contributions

AP and SW were responsible for the study concept and design; made critical revision of the manuscript for important intellectual content. AP was responsible for acquisition, analysis, and interpretation of data and for drafting the manuscript. AP, SW, and DL edited the manuscript.

## Conflict of Interest Statement

The authors declare that the research was conducted in the absence of any commercial or financial relationships that could be construed as a potential conflict of interest.
